# Effects of autologous serum on TREM2 and APOE in a personalized monocyte-derived macrophage assay of late-onset Alzheimer’s patients

**DOI:** 10.1186/s12979-023-00376-2

**Published:** 2023-10-14

**Authors:** Neriman Eren, Susanna Gerike, Berk Üsekes, Oliver Peters, Nicoleta-Carmen Cosma, Julian Hellmann-Regen

**Affiliations:** 1 Department of Psychiatry and Psychotherapy, Charité – Universitätsmedizin Berlin, corporate member of Freie Universität Berlin, Humboldt-Universität Zu Berlin, and Berlin Institute of Health, Section Clinical Neurobiology, Campus Benjamin Franklin, Hindenburgdamm 30, 12203 Berlin, Germany; 2 German Center for Mental Health (DZPG) Partner Site Berlin, Berlin, Germany; 3 BIH Biomedical Innovation Academy, BIH Charité Clinician Scientist Program, Berlin Institute of Health at Charité – Universitätsmedizin Berlin, Charitéplatz 1, Berlin, 10117 Germany

**Keywords:** Late-onset Alzheimer's disease, TREM2, APOE, Monocyte-derived macrophages, Amyloid-beta uptake, Sex differences, Patient-derived personalized assay, Inflammaging

## Abstract

**Background:**

Age-associated deterioration of the immune system contributes to a chronic low-grade inflammatory state known as “inflammaging” and is implicated in the pathogenesis of late-onset Alzheimer's disease (LOAD). Whether changes in the tissue environment caused by circulatory factors associated with aging may alter the innate immune response is unknown. Monocyte-derived macrophages (Mo-MФs) infiltrating the brain alongside microglia are postulated to play a modulatory role in LOAD and both express triggering receptor expressed on myeloid cells 2 (TREM2). Apolipoprotein E (APOE) acts as a ligand for TREM2, and their role in amyloid beta (Aβ) clearance highlights their importance in LOAD. However, the influence of the patient's own milieu (autologous serum) on the synthesis of TREM2 and APOE in infiltrating macrophages remains unknown.

**Objectives:**

To functionally assess patient-specific TREM2 and APOE synthesis, we designed a personalized assay based on Mo-MФs using monocytes from LOAD patients and matched controls (CO). We assessed the influence of each participant’s own milieu, by examining the effect of short- (1 day) and long- (10 days) term differentiation of the cells in the presence of the donor´s autologous serum (AS) into M1-, M2- or M0-macrophages. Additionally, sex differences and Aβ-uptake ability in short- and long-term differentiated Mo-MФs were assessed.

**Results:**

We showed a time-dependent increase in TREM2 and APOE protein levels in LOAD- and CO-derived cells. While AS did not differentially modulate TREM2 compared to standard fetal calf serum (FCS), AS decreased APOE levels in M2 macrophages but increased levels in M1 macrophages. Interestingly, higher levels of TREM2 and lower levels of APOE were detected in female- than in male- LOAD patients. Finally, we report decreased Aβ-uptake in long-term differentiated CO- and LOAD-derived cells, particularly in APOEε4(+) carriers.

**Conclusions:**

We demonstrate for the first time the suitability of a personalized Mo-MФ cell culture-based assay for studying functional TREM2 and APOE synthesis in a patient's own aged milieu. Our strategy may thus provide a useful tool for future research on diagnostic and therapeutic aspects of personalized medicine.

**Supplementary Information:**

The online version contains supplementary material available at 10.1186/s12979-023-00376-2.

## Introduction

Late-onset Alzheimer's disease (LOAD), an age-associated neurodegenerative disorder, is the most prevalent form of dementia [1]. The etiology and pathogenic mechanisms of LOAD are multifactorial [2, 3], with neuroinflammation playing a prominent role [4, 5]. Aging has been associated with increased baseline inflammation, which might contribute to Alzheimer´s disease (AD) pathology [6–8]. A mechanism contributing to pathological brain aging and neurodegeneration may be related to the age-related infiltration of the brain with myeloid cells [9–11]. Brain-infiltrating monocyte-derived macrophages (Mo-MФs) have been found to be associated with Aβ plaques [11, 12] and perform functions similar to those of microglia [12, 13]. Therefore, understanding the role of Mo-MФs in the crosstalk between peripheral and central compartments during physiological and pathological aging could shed light on the development of new therapeutic strategies in AD.

Rare mutations in the gene encoding the triggering receptor expressed on myeloid cells 2 (TREM2) protein are associated with an increased risk for LOAD [14, 15], comparable to mutations in the gene encoding apolipoprotein E (APOE) [16]. APOE acts as a ligand for TREM2 [17], and TREM2-APOE interaction has been shown to be important in modulating AD progression [18, 19]. Genetic modification resulting in increased TREM2 function in mice determined an upregulation of the protective disease-associated microglia (DAM) signature [20], and pointed to the protective function of TREM2-expressing macrophages in the central nervous system (CNS) [21]. Moreover, brain infiltration of TREM2-expressing macrophages has been found both in AD-transgenic mice [11] and postmortem AD-patients [9]. Consistently, increased soluble TREM2 (sTREM2), a proteolytic cleavage product of TREM2, in human cerebrospinal fluid (CSF) with aging and blood–brain barrier (BBB) breakdown provides further insight into the role of TREM2 as a macrophage activation marker [22, 23]. Together, these findings indicate that upregulated TREM2 synthesis might be a conserved macrophage response during tissue damage, including AD pathology. Therefore, investigating personalized modulation of TREM2 as a functional proxy for brain-infiltrating macrophages may provide valuable insights into potential personalized therapeutic interventions in aged individuals [24].

Microglia as well as monocyte-derived macrophages (Mo-MФs) can adopt distinct pro-M1 and anti-M2 inflammatory phenotypes in response to environmental stimuli [24, 25]. The role of the microenvironment and TREM2 in macrophage function was noted by Seno et al. in a study that showed impaired macrophage activation and reduced wound healing in control vs. TREM2-deficient mice following IL-4 and IL-13 neutralization [26]. Consistently, in vitro stimulation of mouse macrophages with M-CSF, IL-4, and IL-13 was shown to activate M2 macrophages, followed by an increase in TREM2 [27–29]. Moreover, M2 differentiated macrophages have also been shown to modulate APOE secretion [30]. We previously reported that M2- and M0- macrophages derived from AD-derived cells exhibit increased TREM2 synthesis when differentiated for the appropriate duration (e.g.,10 days) [24]. Thus, it can be inferred that transitioning the macrophage phenotype toward a neuroprotective M2 state may greatly enhance TREM2 and APOE synthesis in AD patients. While replicating the multifactorial environment of human macrophages in vitro is challenging, assessing Mo-MФ differentiation profiles at the individual patient level is valuable, especially in aged individuals [31].

Differentiation of macrophages is modulated by both intrinsic factors related to age-associated defects in the immune response and extrinsic factors related to age-associated changes in the tissue environment [32, 33]. Hormonal and metabolic changes [34, 35], chronic and latent viral infections [36] and damage-related molecular patterns (DAMPs) [37] can modify the serum composition and hence may impact macrophage differentiation. In elderly populations, a notable change in serum composition is the elevation of inflammatory markers, including C-reactive protein (CRP), interleukin-6 (IL-6) and tumor necrosis factor-alpha (TNF- α) [38, 39]. This chronic low-grade inflammation is known to hyperactivate monocytes and macrophages during disease progression in AD [40]. In addition to aging, the sex differences in serum composition due to differential regulation of sex steroids [41], gut microbiota composition [42, 43] and/or genetic/epigenetic factors [44] may cause differences in macrophage response between males and females. Since these dynamic changes are hypothesized to occur in a highly patient-specific manner, our main strategy was to capture the patients’ own milieu by preserving their autologous sera (AS), which should closely mimic each patient’s in vivo milieu. By first separating cells from their sera and later (re-) exposing them in a very systematic, defined manner to the respective matching AS, namely their former milieu, we sought to characterize an individual contribution of the donor’s milieu to macrophage function in vitro.

Due to the significant influence of the microenvironment on macrophage response [45], we asked whether the macrophage responses we previously observed in AD-derived cells under standard fetal calf serum (FCS) [24] could be reproduced in more physiologically relevant conditions in a second patient cohort. To this end, we differentiated monocytes from aged LOAD patients and matched controls into M1-, M2- or M0- macrophages in the presence of donors´ AS by using our well-established patient-specific Mo-MФ differentiation assay [24, 31]. We assessed the modulation of TREM2 and APOE synthesis in short-(1 day) and long-term-(10 days) differentiated patient-specific Mo-MФs in AS and the Aβ-uptake. Finally, we also considered the potential consequences of sex and the APOEε4 genotype effects.

## Material and methods

### Participant recruitment and study design

Patients with a clinically confirmed diagnosis of AD (*n* = 21) and healthy controls (CO, *n* = 16) matched for age, APOE status and body mass index (BMI) were included in the study (Table 1). All enrolled participants were > 65 years of age and signed an informed consent from approved by the local ethics committee (EA4/002/13). The diagnostic criteria of AD were defined according to the 2011 National Institute on Aging (NIA) – Alzheimer's Association Diagnostic Guidelines; all AD patients had a confirmed CSF diagnosis of AD according to the A + T + N + criteria [46, 47]. Healthy controls with confirmed CSF A-T-N- were recruited through the memory clinic of the department. Participants received clinical evaluation, physical examination, blood and CSF sampling, and medical history assessment. Exclusion criteria for all participants were any clinical signs of acute inflammation, recent infection requiring antibiotics (< one month), recent vaccination (< 3 months), chronic inflammatory disease, heart failure, clinically manifested asthma or allergies, history of cancer and stroke, pregnancy and lactation as well as poorly controlled diabetes, cardiovascular, renal, hepatic, hematologic, endocrine and neurologic disease, and chronic use of nonsteroidal anti-inflammatory drugs or corticosteroids. All procedures complied with the ethical standards of national and institutional committees on human experimentation and with the Helsinki Declaration of 1975, as revised in 2013 [48].

**Table 1:** Characteristic of participants

	**AD (*n* = 21)**	**CO (*n* = 16)**	***p*-value**
Age	72.76 (7.61)	74.93 (6.56)	0.654
BMI (kg/m^2^)	22.85 (3.73)	22.08(2.8)	0.673
Gender (m/f)	10/11	5/11	> 0.999
APOE ε4 status (pos. /neg.)	10/11	6/10	> 0.999
MMSE score	24.13 (3.45)	27.94 (2.51)	**0.0004**
CSF Aβ_1–42_	455 (239.13)	952.19 (375.2)	** < 0.0001**
CSF Aβ_1-42_ /Aβ_1–40_	0.048 (0.021)	0.091 (0.02)	** < 0.0001**
CSF – p-tau_181_	114.82 (52.98)	39.45 (10.94)	** < 0.0001**
CSF – t-tau	699.32 (274.52)	279.69 (79.23)	** < 0.0001**

Data are shown as mean and standard deviation (SD) unless otherwise stated. *AD *Alzheimer´s disease, *CO *Control, *APOE *Apolipoprotein, *CSF *Cerebrospinal fluid, *Aβ *Amyloid β peptide, *MMSE *Mini-mental state examination, *P-tau*_181_ tau phosphorylated at threonine 181, *T *Tau-total tau. Probability values (p) denote differences between groups. Fisher's Exact Tests performed to compare gender and APOEε4 differences between groups. Pairwise comparison of groups was performed with Mann Whitney tests (unpaired groups)

### Sample collection and isolation of PBMCs

Peripheral blood mononuclear cells (PBMCs) were obtained from heparinized venous blood by FICOLL™ density gradient centrifugation using established protocols [24, 31, 49]. The separated PBMC layers were washed twice with cold PBS and then stored at -80 °C until further use. Serum samples were collected in vacuum extraction tubes (BD Biosciences, Germany), allowed to clot at room temperature (RT) and then extracted by centrifugation at 2500 rpm for 5 min at RT. The extracted serum samples were stored at -80 °C until use to produce an RPMI-based culture medium containing 10% AS and 1% penicillin/streptomycin (P/S) [31, 50].

### Monocyte enrichment and differentiation to Mo-MФs

**Fig. 1:**
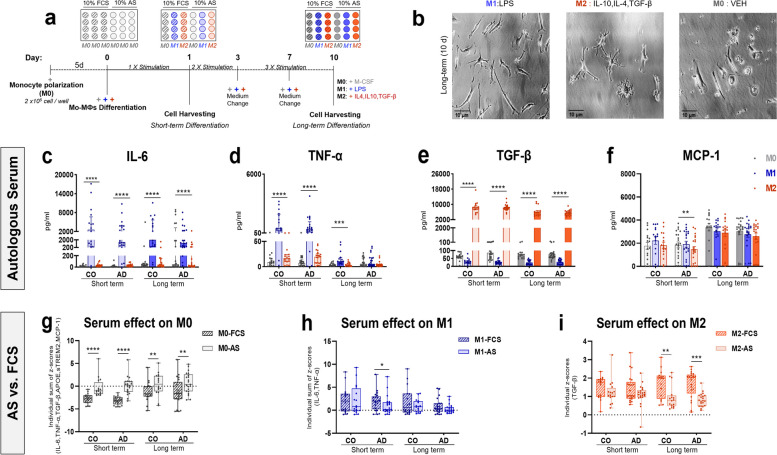
Short- and long-term differentiation of patient-derived M0-, M1- and M2- macrophages in autologous serum (AS). **a** Experimental design: Mo-MФs were differentiated for the total periods of 6 and 15 days with M-CSF, of which respectively the last 1 (short-term) and 10 days (long-term) were under differentiation to M1 (LPS), M2 (IL-4, IL10, TGF-β) and M0 (vehicle) macrophages in AS or FCS. **b** Morphology of the Mo-MФs after long-term differentiation in AS observed with a Zeiss Axiovert 10 inverted microscope and captured with a SWIFT Cam SC500 5.1 MP. Magnification = 25x, scale bar = 10 µm. Secretion levels of **c** IL-6 and **d** TNF-α; **e** TGF-β and **f** MCP-1 inflammatory markers in short-term and long-term differentiated Mo-MФs from patients with AD (*n* = 21) and CO (*n* = 16) in AS. Effect of AS vs. FCS on short-term and long-term **g** M0, **h** M1 and **i** M2 differentiated macrophages from patients with AD (*n* = 21) and CO (*n* = 16). Dots represent individual participant values. **c-f** Closed bars and symbols represent M0 (light grey for short-term; dark grey for long-term), M1 (light blue for short-term; dark blue for long-term) and M2 (light orange for short-term; dark orange for long-term) macrophages respectively. **g-i** Closed bars and symbols represent M0 (grey with pattern for FCS; without pattern for AS), M1 (blue with pattern for FCS; without pattern for AS) and M2 (orange with pattern for FCS; without pattern for AS) macrophages respectively. The Friedman ANOVA test or Wilcoxon signed rank test (paired) was used to compare within-group differences. Significant values of ANOVA tests were then subjected to Dunn's multiple comparison test with Bonferroni correction. Statistical significance was determined at the *p* ≤ 0.05 level unless a Bonferroni adjustment was required for multiple comparisons (*p* = 0.0167 (p/n, assuming *n* = 3 comparisons))

Human PBMCs were cultured as described previously [24, 31]. Briefly, isolated PBMCs were cultured in RPMI 1640 medium (Biochrom,Berlin, Germany) supplemented with 1% P/S (10,000 U/10 mg per ml; Biochrom, Germany) in a 24-well plate (2 × 10^5^ cell/well) at 37 °C with 5% CO_2_. Cell culture medium containing human macrophage colony-stimulating factor (M-CSF) (10 ng/ml) (Miltenyi Biotec, Germany) was supplemented with one of the following: 10% fetal calf serum (FCS, Biochrom, Germany) or 10% autologous serum (AS) from the respective donor (Fig. 1a). After overnight incubation, half of the medium was replaced with fresh RPMI 1640 medium containing human M-CSF (10 ng/ml), 1% P/S and 10% FCS or 10% AS. All nonadherent cells were removed by replacing the culture medium with fresh medium on day 2 of plating. Attached monocytes were polarized to macrophages for a total period of 5 days with M-CSF, followed by 1 day (short-term, 1 × stimulation) and 10 days (long-term, 3 × stimulation) of differentiation to M1 (50 ng/ml LPS, Sigma-Aldrich, USA), M2 (IL-4, IL-10, TGF-β each 20 ng/ml, Peprotech, USA) according to previously published protocols or left as M-CSF stimulated M0-macrophages (vehicle) (Fig. 1a; See Supplementary Table 1 for lot numbers) [31]. Each stimulation condition was performed in duplicate, and fresh media exchange (25%) was performed on days 3 and 7 of differentiation. Cell and supernatant samples were collected 1 day and 10 days after Mo-MФ differentiation and stored at -80 °C for further measurements.

### Neuroinflammatory marker and APOE synthesis in Mo-MФ cultures

Extracellular secretion levels of pro- IL-6 (interleukin 6), TNF-α (tumor necrosis factor alpha) and anti- TGF-β1 (transforming growth factor beta β1) inflammatory cytokines, MCP-1 (monocyte chemoattractant protein-1) chemokine, sTREM2 (soluble TREM2) and APOE (apolipoprotein E) levels were measured in the Mo-MФ culture supernatant and the patient serum samples using the LEGENDplex™ Human Neuroinflammation Panel (BioLegend, San Diego, USA) and LEGENDplex™ Human Apolipoprotein (Apo) Panel (a single-plex: APOE) (BioLegend, San Diego, USA) according to the manufacturer's instructions. Data were acquired using the BD FACS Canto™ II (Biosciences), and analysis was performed using LEGENDplex Data Analysis Software v8.0 (BioLegend, San Diego, USA) by the manufacturer’s instructions. Detection limit ranges (pg/ml) for neuroinflammatory markers and APOE are shown in Supplementary Table 2. All measurements were performed in parallel, and both cell culture media and serum samples from AD and CO were quantified on the same plates. Replicates were quantified with coefficient variants (CVs) less than 30% as recommended by the manufacturer. To assess the serum effect, protein levels in the cell culture supernatant of M1 and M2 macrophages were normalized to those in M0-(vehicle) macrophage supernatants [51].

### Total RNA extraction and TREM2 expression analysis

TRI Reagent ™ (Zymo-Research, Germany) was used to extract total RNA from Mo-MФs according to the manufacturer's instructions, and extracted RNA was stored at -80 °C for further measurement. cDNA was synthesized from total RNA by following the standard protocol of the Revert Aid First Strand cDNA Synthesis Kit™ (Thermo Fisher Scientific Inc., MA, USA) and was stored at -20 °C until use. Real-time quantitative polymerase chain reaction (RT-qPCR) was performed using Applied Biosystems StepOne™ Real-Time PCR System (CA, USA) using the LightCycler™ 480 SYBR Green I Master (Roche, Mannheim, Germany). The RT-qPCR reactions of the samples were run in duplicate. GAPDH (F: TTG CCA TCA ATG ACC CCT TCA; R: CGC CCC ACT TGA TTT TGG A) was used as the housekeeping gene for data normalisation (ΔCt) and the relative expression level of TREM2 (F: CAG CCA TCA CAG ACG ATA CCC; R: AAG TGG GTG GGA AGG GGA TTT C) was determined by the 2^−ΔCt^ method.

### Human Aβ_1-42_ uptake in in vitro short- and long-term M1- and M2- macrophage cultures

Monocytes were isolated and differentiated into M1 (LPS) and M2 (IL-4, IL-10, TGF-β) macrophages in a 24-well plate as described above. Cells were cultured in RPMI 1640 medium containing 10% FCS and 1% P/S with human M-CSF (10 ng/ml). To measure Aβ-uptake capacity, macrophages were incubated with 50 nM human Aβ_1-42_ (BioLegend, San Diego, USA, Lot: B225454) for 24 h at 37 °C following 1 day (short-term) and 10 days (long-term) of macrophage differentiation. Mo-MФs were washed with phosphate-buffered saline (PBS) and lysed with protein lysis buffer following a previously published protocol [24]. Uptake of human Aβ_1-42_ peptide in M1- and M2- macrophage cultures was measured with Human Neurodegenerative Biomarker Panel 1 (a single-plex: Aβ_42_) (BioLegend, San Diego, USA). Measured Aβ-uptake levels in cell lysates were normalized by total protein concentration assessed by bicinchoninic acid** (**BCA) protein assay (Thermo Scientific Pierce BCA protein assay reagents, MA, USA) [24].

### Confocal microscopy imaging of Aβ_1-42_ uptake

To observe Aβ-uptake in M1- and M2- macrophage cultures, monocytes were plated on polylysine-coated coverslips in a 12-well plate (5 × 10^5^ cells/well) and differentiated into Mo-MФs as described above. Following 1 day (short-term) and 10 days (long-term) of M1- and M2-differentiation in FCS culture conditions, cells were incubated with 500 nM human FAM-Aβ_1‐42_ (AnaSpec, United States, Lot: 1,758,517) for 24 h at 37 °C. For the last 30 min of Aβ-uptake, cell nuclei were stained with Hoechst (1 µg/ml; Lot #21J28) at 37 °C and the unbound dye was removed by washing with PBS. Stained cells were fixed with 4% paraformaldehyde (PFA) and washed twice before mounting on a microscope slide. A 63 × Zeiss Plan-Apochromat 1.4NA oil immersion lens was then used to capture representative images at higher magnification. Image acquisition and analysis were performed using Zeiss Zen and ImageJ software, respectively (Molecular Devices).

### Data analysis

Data are presented as the median ± interquartile range (IQR). GraphPad Prism version 8.0.2 (GraphPad Software, La Jolla, USA) was used for graphic design and statistical analysis. Comparisons of sex differences and APOEε4 status between groups were analysed using Fisher's exact test. The nonparametric The Friedman ANOVA test or Wilcoxon signed rank test (paired) was used to compare within-group differences, while the Kruskal–Wallis test or Mann Whitney U test (unpaired) was used to assess between-group differences. Significant values of analysis of variance (ANOVA) were then subjected to Dunn's multiple comparison tests with Bonferroni correction. Statistical significance was determined at the *p* ≤ 0.05 level unless a Bonferroni adjustment was required for multiple comparisons (where in the case of number (n) of comparisons as performed for M1/M2/M0 was 3, the threshold for significance level was *p* ≤ 0.0167).

To assess the serum differences between AS and FCS in Mo-MФ differentiation from AD- and CO-derived cells, we calculated cumulative scores as described in [31, 52, 53]. Cumulative z-scores for M0, M1 and M2 differentiation were calculated by summing the individual z-scores of inflammatory markers secreted from each participant as detailed in [31], in which the sum of individual IL-6 and TNF-α z-scores for M1, TGF-β z-scores for M2 and IL6-TNF-α, TGF-β, MCP-1, sTREM2 and APOE z-scores for M0 differentiation were used.

To account for inter-assay variation in the performed multiplex assays, we used an approach based on standardization of the plate effect using MFIs of standards with the same concentrations in different plates for the same marker [54]. The modified procedure we proposed to reduce plate variation consists of 5 steps: (1) obtaining linear mean fluorescent intensity (MFI) values for standards and test samples,(2) subtracting the background signal (C0) from standards and test samples in each plate; (3) converting the MFI values to natural log values; (4) calculating the overall average MFI values (corrected MFI) for each concentration of standards in the plates; and (5) determining the protein concentrations of test samples by analysing the log plot using the provided standard concentrations and corrected standard MFIs.

## Results

### Characteristics of participants

The demographic and clinical features of the patients with AD and matched controls (CO) are listed in Table 1. We recruited 37 clinically well-characterized AD patients (*n* = 21, 52% female) and CO (*n* = 16, 68.75% female). Fisher's exact tests did not reveal a difference in sex or APOEε4 allele between CO and AD patients. Furthermore, no difference in age, BMI, or serum inflammatory marker levels between groups was observed. As expected, the MMSE (mini-mental state examination) scores and CSF levels of Aβ_42_ were significantly lower in AD patients than in CO (*p* = 0.0004), whereas CSF levels of T-tau and P-tau were significantly higher (Table 1).

### Short- and long-term differentiation of patient-derived Mo-MФs in autologous sera

Monocytes are known to differentiate into specific macrophage phenotypes based on environmental stimuli [55]. After long-term (10 days) differentiation of AD- and CO-derived cells in AS with LPS (M1), IL-4/ IL-10/ TGF-β (M2) or vehicle (M0), we observed typical M1-, M2- and M0-morphologies (Fig. 1a, b), as previously described in FCS [24].

To assess the effectiveness of M1 and M2 macrophages in AS, we measured the secretion levels of the pro-inflammatory cytokines IL-6 and TNF-α as M1- markers, and the anti-inflammatory cytokine TGF-β as an M2- marker following short-term (1 day) and long-term (10 days) differentiation. As expected, in both short-term and long-term AD- and CO-derived cells, M1 macrophages secreted higher levels of IL-6 and TNF-α, while M2 macrophages exhibited markedly higher levels of TGF-β (Fig. 1c-e; Supplementary Table 3). We also examined the secretion levels of MCP-1, an important chemokine that regulates the capacity of monocytes/macrophages to migrate and infiltrate sites of inflammation. There was a significant decrease in MCP-1 levels in M2- macrophages compared to both M0- and M1- macrophages after short-term differentiation of AD-derived cells only (Fig. 1f; Supplemental Table 3). Together, these results are consistent with our previous findings in FCS [24] and provide further evidence that our patient-specific cell culture assay accurately reflects differences in the inflammatory profiles of Mo-MФs in AS.

### Autologous serum differentially modulates short- and long-term Mo-MФ differentiation

Small molecules circulating in peripheral blood may influence the immunomodulatory activity of mononuclear cells [56]. We assessed the influence of individual AS on short- and long-term Mo-MФ differentiation compared to standard FCS. For this, we calculated the individual cumulative Z-scores of IL-6 and TNF-α for M1- and TGF-β for M2- differentiation in AS- vs. FCS conditions [31]. Since M0 macrophages do not exhibit a distinct cytokine modulation profile, for M0-macrophages we assessed cumulative Z-scores of all measured markers (IL-6, TNF-α, TGF-β, MCP-1, sTREM2, and APOE). M0 macrophages showed significantly higher Z-scores in AS vs. FCS conditions both in AD- and CO-derived cells following short- and long-term differentiation (Fig. 1g, Supplementary Table 4). Moreover, after short-term M1-differentiation, only the Z-score of AD-derived cells was significantly lower in AS than in FCS (Fig. 1h, Supplementary Table 4). There was no serum difference between AS and FCS in Z-scores after short-term M2-differentiation. However, long-term differentiated M2 macrophages in both groups showed significantly lower Z-scores in AS than in FCS (Fig. 1i, Supplementary Table 4).

### Reduced TREM2 modulation in AD-derived Mo-MФs after short-term differentiation

**Fig. 2:**
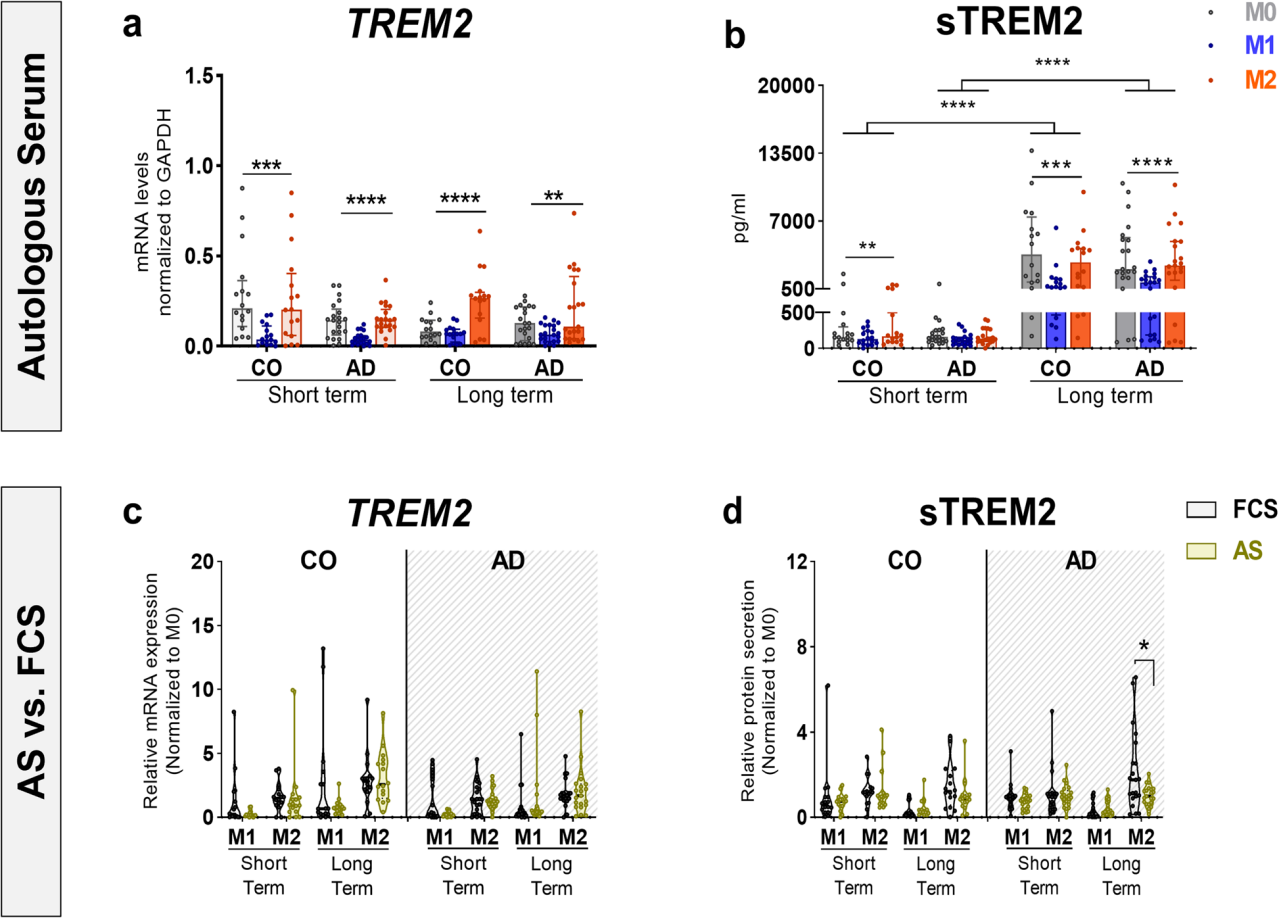
Autologous serum modulation of TREM2 synthesis in short- and long-term differentiated Mo-MФs and serum effect. **a** *TREM2* mRNA and **b** sTREM2 levels in Mo-MФ cultures from AD patients (*n* = 21) and CO (*n* = 16) in AS. Effect of AS vs. FCS on **c** *TREM2* mRNA and **d** sTREM2 levels in short-term and long-term Mo-MФs from AD patients (*n* = 21) and CO (*n* = 16). Dots represent individual participant values. **a**, **b** Closed bars and symbols represent M0 (light grey for short-term; dark grey for long-term), M1 (light blue for short-term; dark blue for long-term) and M2 (light orange for short-term; dark orange for long-term) macrophages respectively. **c**, **d** Closed violins and symbols represent AS (green) and FCS (dark grey). mRNA (normalized to GAPDH) expression was measured with RT-qPCR. The Friedman ANOVA test or Wilcoxon signed rank test (paired) was used to compare within-group differences. Significant values of ANOVA tests were then subjected to Dunn's multiple comparison test with Bonferroni correction. Statistical significance was determined at the *p* ≤ 0.05 level unless a Bonferroni adjustment was required for multiple comparisons) p* < 0.0167 (p/n, assuming *n* = 3 comparison)

We previously reported a patient-specific TREM2 modulation in short- and long-term differentiated M0, M1 and M2 macrophages in the FCS condition [24]. However, it is unknown whether this modulation is mediated through patient-specific circulating factors that are present in AS. Therefore, we examined the influence of aging macrophages' own milieu on TREM2 mRNA (*TREM2*) and soluble TREM2 (sTREM2) synthesis, by differentiating patient-specific Mo-MФs in the AS condition. We did not observe a difference in TREM2 synthesis between CO- and AD-derived Mo-MФs in the presence of AS (Fig. 2a, b). Moreover, there was no sex or APOEε4 genotype effect on TREM2 synthesis across groups (Supplementary Fig. 1 c-f). After short-term differentiation (1 day) in AS, CO-derived cells revealed higher *TREM2* mRNA and sTREM2 levels in M0- and M2- macrophages than M1-macrophages (Fig. 2a, b, Table 2). However, AD-derived cells showed an increase only in *TREM2* mRNA levels in M0- and M2- macrophages after short-term differentiation in AS (Fig. 2a, b, Table 2).

**Table 2:** Modulation of TREM2 and APOE levels in short- and long-term differentiated Mo-MФs in autologous serum (AS)

	**SHORT-TERM**							**LONG-TERM**						
**CO (*n* = 16)**														
Gene*/ **Protein**	Friedman statistic	*p*-value	Dunn´s multiple comparison tests					Friedman statistic	*p*-value	Dunn´s multiple comparison tests				
			M1 vs. M2		M1 vs. M0		M2 vs. M0			M1 vs. M2		M1 vs. M0		M2 vs. M0
*TREM2*	16.63	0.0002	**0.0080**		**0.0003**		> 0.9999	21.5	< 0.0001	** < 0.0001**		0.2313		**0.0140**
sTREM2	9.875	0.0072	0.0647		**0.0080**		> 0.9999	17.38	0.0002	**0.0140**		**0.0001**		0.6478
APOE	0.1250	0.9394	> 0.9999		> 0.9999		> 0.9999	0.875	0.6456	> 0.9999		> 0.9999		> 0.9999
**AD (*n* = 21)**														
*TREM2*	22.57	< 0.0001	** < 0.0001**	**0.0036**		0.4947		11.81	0.0027	**0.0021**	0.0923		0.6511	
sTREM2	6	0.0498	0.1922	0.0619		> 0.9999		20.86	< 0.0001	**0.0006**	** < 0.0001**		> 0.9999	
APOE	0.2857	0.8669	> 0.9999	> 0.9999		> 0.9999		2.571	0.2765	0.4947	> 0.9999		0.4947	

qPCR products* (in italic) and proteins calculated using Friedman ANOVA Test with significance values adjusted by the Bonferroni correction (*p* < 0.0167) for Dunn´s multiple comparison tests for all measured markers in M1. M2. and M0 after short- and long-term differentiation. TREM2- triggering receptor expressed on myeloid cells 2; sTREM2- soluble triggering receptor expressed on myeloid cells 2; APOE- apolipoprotein E

To evaluate serum differences in patient-specific TREM2 levels between AS- and FCS- conditions, we normalized M1- and M2- macrophage to M0 (vehicle)- macrophage data to eliminate possible serum contributions. We observed comparable levels of *TREM2* mRNA and sTREM2 between AS- and FCS- conditions after short-term M1- and M2- differentiation (Fig. 2c, d, Supplementary Table 5).

### Long-term M0- and M2- differentiation enhances TREM2 synthesis in AD-derived cells

After long-term differentiation (10 days), in contrast to short-term differentiation (1 day), not only CO- but also AD-derived M0- and M2- macrophages significantly upregulated sTREM2 levels in AS (Fig. 2b, Table 2). Moreover, CO-derived long-term M2 macrophages showed a significant increase in *TREM2* mRNA levels compared to M0- and M1-macrophages (Fig. 2a, Table 2). However, AD-derived long-term M2 macrophages exhibited significantly higher *TREM2* mRNA levels only compared to M1 macrophages. Accordingly, AD-derived long-term M0 macrophages showed comparable levels to M2 and expressed higher *TREM2* mRNA levels than M1, albeit not significant (Fig. 2a, Table 2). Together, these findings in AS condition are consistent with our previous findings in FCS condition [24], and demonstrate that differentiation of Mo-MФs into M2-macrophages and unstimulated M0- macrophages for an appropriate period can upregulate sTREM2 and *TREM2* mRNA levels in patients with AD not only in FCS but also in AS.

We, next, analysed differences in TREM2 levels in AS vs. FCS after long-term Mo-MФ differentiation. Our findings showed a significant difference in sTREM2 levels but not in *TREM2* mRNA expression levels (Fig. 2c, d, Supplementary Table 5). Notably, long-term M2 macrophages derived from AD exhibited lower sTREM2 levels in AS than in FCS.

### Autologous serum differentially modulates APOE secretion after long-term differentiation

**Fig. 3:**
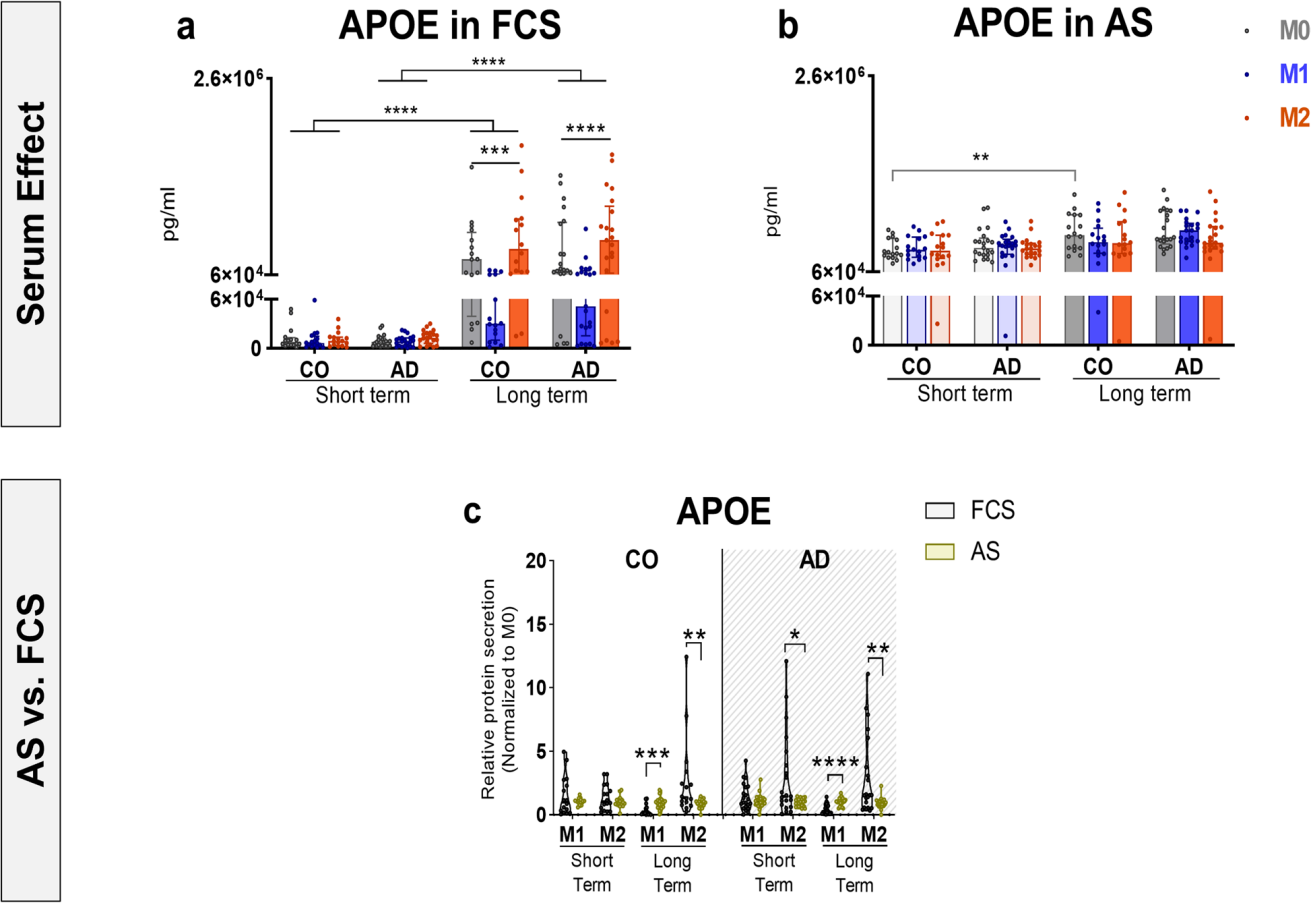
APOE synthesis in short- and long-term differentiated Mo-MФs and serum effects. APOE secretion levels in Mo-MФs cultures from AD patients (*n* = 21) and CO (*n* = 16) **a** in FCS or **b** in AS. **c** Effects of AS vs. FCS on APOE secretion levels in short-term and long-term Mo-MФs from AD patients (*n* = 21) and CO (*n* = 16). Dots represent individual participant values. **a**, **b** Closed bars and symbols represent M0 (light grey for short-term; dark grey for long-term), M1 (light blue for short-term; dark blue for long-term) and M2 (light orange for short-term; dark orange for long-term) macrophages respectively. **c** Closed violins and symbols represent AS (green) and FCS (dark grey). mRNA (normalized to GAPDH) expression was measured with RT-qPCR. The Friedman ANOVA test or Wilcoxon signed rank test (paired) was used to compare within-group differences. Significant values of ANOVA tests were then subjected to Dunn's multiple comparison test with Bonferroni correction. Statistical significance was determined at the *p* ≤ 0.05 level unless a Bonferroni adjustment was required for multiple comparisons) p* < 0.0167 (p/n, assuming *n* = 3 comparison)

Given the protective role of APOE as a TREM2 ligand [57], we aimed to look at the APOE synthesis in our patient-specific cell culture assay for the first time. We did not observe a difference in APOE levels between CO- and AD-derived cells under either AS- or FCS- conditions (Fig. 3a, b). In addition, there was no APOEε4 genotype or sex effect on APOE levels across the groups (Supplementary Fig. 2a-d). After short-term differentiation in AS- or FCS- conditions, Mo-MФs showed comparable levels of APOE secretion in both groups (Fig. 3a, b). When the cells were long-term differentiated in FCS, both AD- and CO-derived M2 macrophages revealed a significant increase in APOE levels compared to M1 macrophages, while long-term M0 macrophages showed a significant increase only in AD-derived cells (Fig. 3a, p_CO-M2vsM1_ = 0.003, p_AD-M2vsM1_ =  < 0.0001 p_AD-M0vsM1_ = 0.0012). However, in contrast to FCS, Mo-MФs showed comparable levels of APOE secretion in both groups when differentiated long-term in AS (Fig. 3b, Table 2). These results suggest a regulatory role for patient-specific circulating factors in AS for APOE synthesis.

We further evaluated differences between AS- and FCS- conditions in APOE secretion and found significantly lower APOE in AS- than in FCS- only in short-term differentiated AD-derived M2 macrophages (Fig. 3c, Supplementary Table 5). When the cells were long-term differentiated, however, both CO- and AD-derived M2 macrophages showed lower APOE levels in AS compared to FCS, whereas M1 macrophages exhibited higher secretion levels (Fig. 3c, Supplementary Table 5).

### Sex differences in fold change in TREM2 and APOE synthesis in senescent Mo-MФs in vitro

**Fig. 4:**
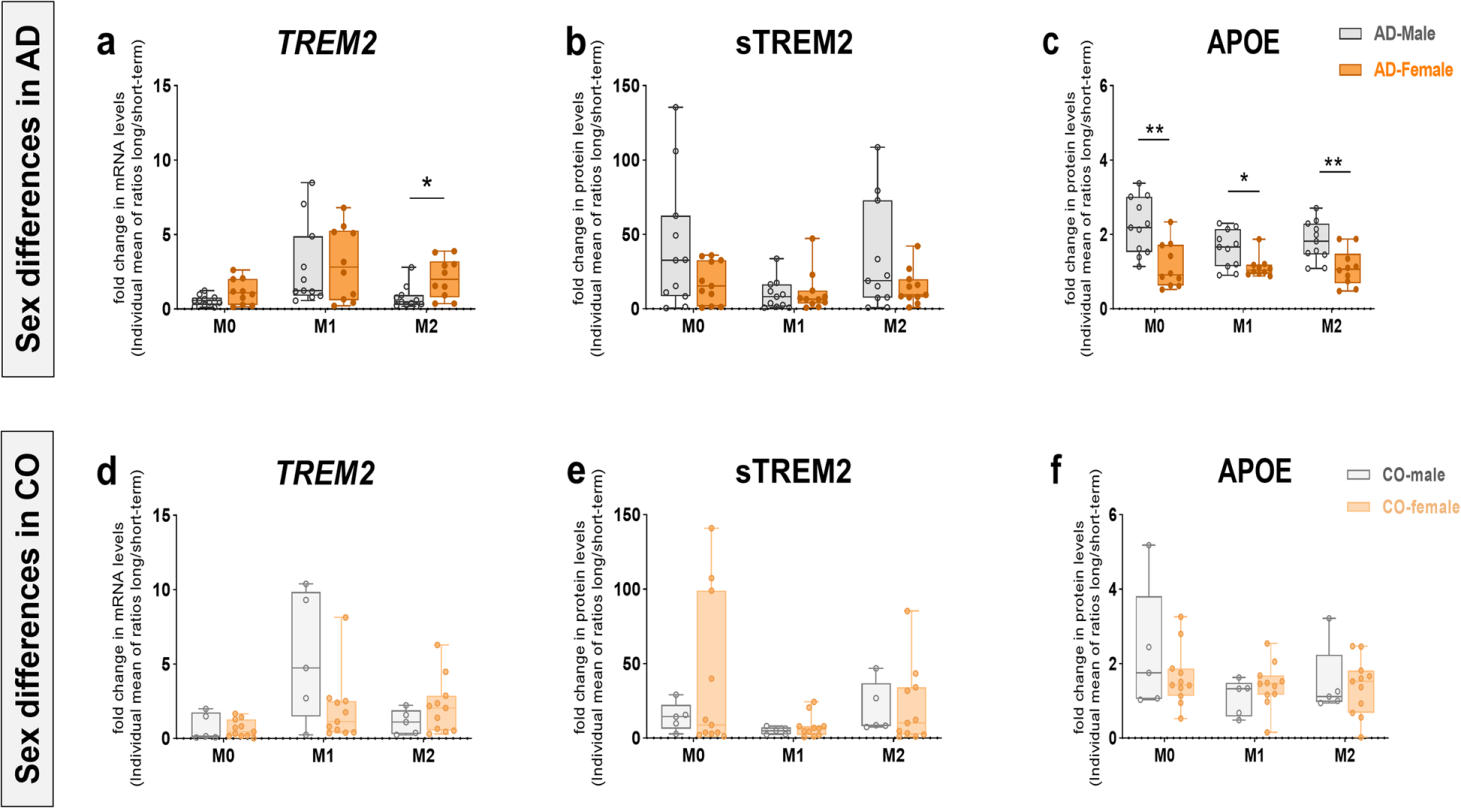
Sex differences in TREM2 and APOE levels in patient-specific Mo-MФs. Fold increase in **a** *TREM2* mRNA, **b** sTREM2 and **c** APOE levels in Mo-MФ cultures from AD-female patients (*n* = 10) vs. AD-male patients (*n* = 11) after long-term compared to short-term differentiation in AS. Fold increase in **d** *TREM2* mRNA, **e** sTREM2 and **f** APOE levels in Mo-MФs cultures from CO-female patients (*n* = 11) vs. CO-male patients (*n* = 5) after long-term compared to short-term differentiation in AS. **a-f** Closed bars and circle symbols represent male (dark grey for AD, light grey for CO) or female (dark orange for AD, light orange for CO) patients. mRNA (normalized to GAPDH) expression was measured with RT-qPCR. A pairwise comparison of groups was performed with the Mann Whitney U-test (paired groups) (*p < 0.05, **p < 0.01, ***p < 0.001)

The increased synthesis of TREM2 and APOE in AD-derived senescent Mo-MФs after long-term differentiation (10 days) (Figs. 2a, b; 3b; Supplementary Table 6) prompted us to evaluate a possible association of sex. To assess the extent of sex-associated changes in TREM2 and APOE levels after long-term differentiation, we calculated the individualized ratios of mRNA or protein levels in long- to short-term Mo-MФ cultures in AS (Fig. 4a-f). There was no sex difference in TREM2 and APOE levels between the AD and CO groups (Supplementary Fig. 3). We found a sex difference in the fold change in TREM2 and APOE levels in AD-derived cells only (Fig. 4 a, c, Supplementary Table 7). After long-term differentiation, there was a significant fold increase in *TREM2* mRNA levels in M2 macrophages from female-derived cells compared to those from male-derived cells (Fig. 4a, U = 21 *p* = 0.0156). Moreover, female-derived cells showed a higher fold increase, albeit nonsignificant, in *TREM2* mRNA in M0 macrophages (*p* = 0.13), while M1 macrophages showed comparable levels of fold change with male-derived cells (Fig. 4a, Supplementary Table 7). In contrast to transcript levels, higher fold changes in sTREM2 and APOE protein levels were observed in male- compared to female-derived M0, M1 and M2 macrophages (Fig. 4b, c; U_APOE/M0_ = 17 p_APOE/M0_ = 0.0062, U_APOE/M1_ = 25 p_APOE/M1_ = 0.0357, U_APOE/M2_ = 18 p_APOE/M2_ = 0.0079). However, the increase in sTREM2 levels was not statistically significant (Fig. 4b, Supplementary Table 7).

### Reduced Aβ uptake capacity after long-term M1- and M2- differentiation

**Fig. 5:**
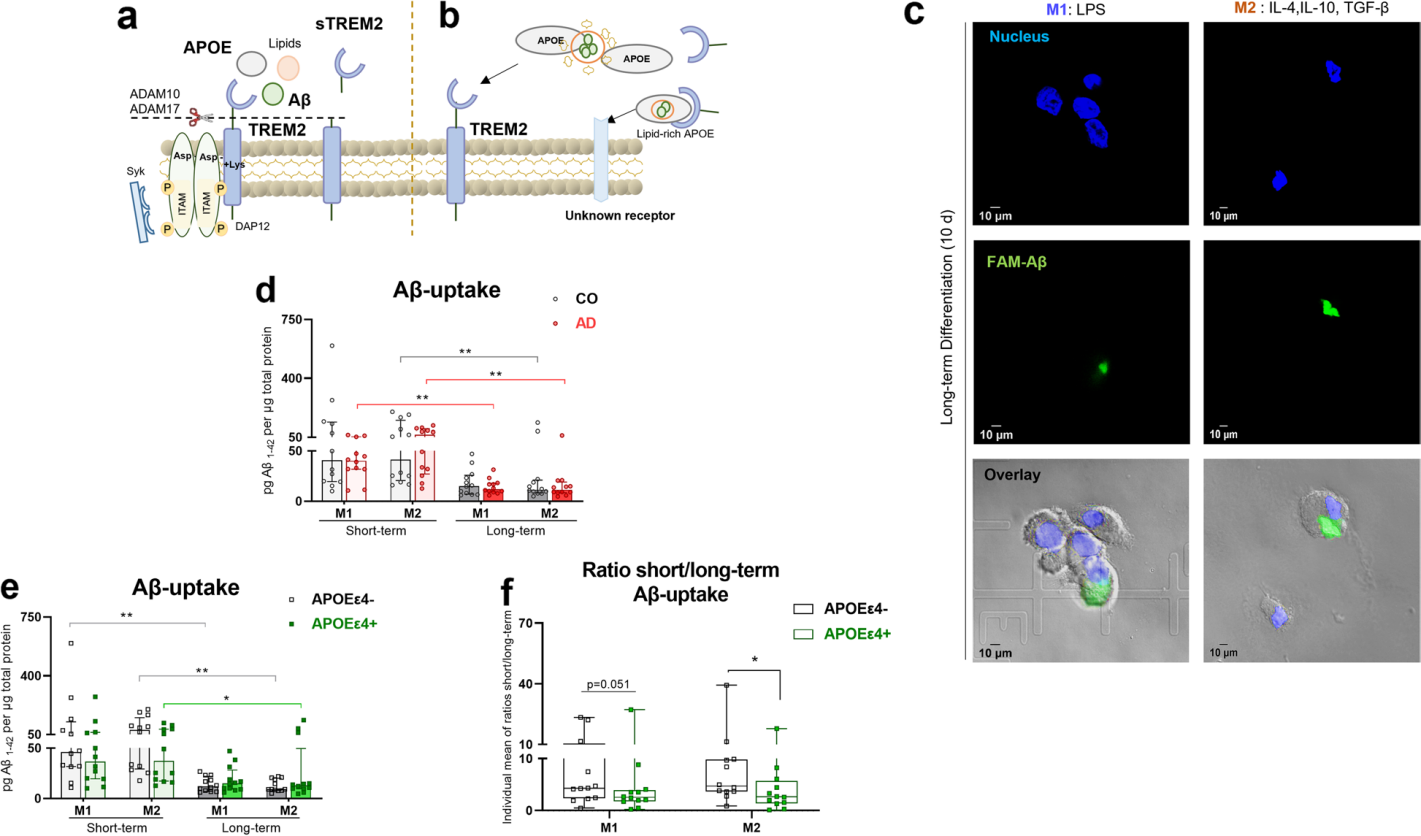
Lower Aβ-uptake of APOEε4( +)- derived M1- and M2- macrophages. **a**, **b** Schematic overview of TREM2 proteolytic cleavage product sTREM2 formation and TREM2-modulated Aβ uptake. **c** Confocal microscopy analysis of fluorescence Aβ (green)-uptake by long-term differentiated M1- and M2- macrophages in FCS at 24 h, representative of 3 independent experiments. Staining for the nucleus (blue) shows that Aβ_1-42_ peptides are internalized within the cells. Magnification = 63x, scale bar = 10 µm. Comparison of Aβ-uptake ability of M1- and M2- differentiated macrophages in FCS from patients with **d** AD vs. CO (*n* = 12, per group) and **e** APOEε4(+) and APOEε4(-) (*n* = 12, per group). **f** Genotype effect on the fold change in Aβ-uptake ability due to long-term compared to short-term M1-and M2- macrophage differentiation in FCS. Aβ-uptake levels in Mo-MФs were quantified with a bead-based immunoassay. Dots represent individual participant values (circle for AD and CO; square for APOEε4(+) and APOEε4(-)). Closed bars and symbols represent CO (light grey for short-term; dark grey for long-term) vs. AD (light red for short-term; dark red for long-term) and APOEε4(-) (light grey for short-term; dark grey for long-term) vs. APOEε4(+) (light green for short-term; dark green for long-term). Open bars and symbols represent APOEε4(-) (black), while open bars and closed symbols represent APOEε4(+) (green). The Friedman ANOVA was used to compare within-group differences, while the Mann Whitney U-test (paired groups) was used to assess between-group differences. Significant values of ANOVA tests were then subjected to Dunn's multiple comparison test with Bonferroni correction. Statistical significance was determined at the *p* ≤ 0.05 level unless a Bonferroni adjustment was required for multiple comparisons (p* < 0.0167 (p/n, assuming *n* = 3 comparison)

Given the importance of the TREM2-APOE-interaction in Aβ pathology [58, 59], we examined whether TREM2/APOE modulation is associated with the Aβ-uptake capacity of Mo-MФs, (Fig. 5a, b). We did not include unstimulated-M0 macrophages, since Aβ plaque-associated macrophages were proposed to be in an activated state. Furthermore, all the conditions were carried out in the FCS culture condition to assess the macrophages' Aβ-uptake capacity, regardless of the effects of circulating factors in AS. Figure 5c shows internalized Aβ aggregates in M1- and M2-macrophages incubated for 24 h with fluorescent Aβ following long-term differentiation. In parallel, we quantified the in vitro Aβ-uptake ability of M1- and M2-macrophages by quantifying Aβ in cell lysates using a traditional, highly sensitive bead-based immunoassay. The data revealed comparable uptake levels between CO- and AD-derived cells after both short- and long-term differentiation. (Fig. 5d). There was no sex or genotype difference in Aβ-uptake in the groups (Supplementary Fig. 4). We found reduced Aβ uptake ability in M2 macrophages derived from both AD- and CO-derived cells after long-term differentiation, whereas the reduction in M1 macrophages was only seen in AD-derived cells (Fig. 5d, p_CO-M2shortvslong_ = 0.0089, p_AD-M2shortvslong_ = 0.001 in M2 macrophages; p _AD-shortvslong_ = 0.0089 in M1 macrophages).

Independent of disease status, we also assessed the effect of the APOEε4 genotype on the Aβ uptake ability of Mo-MФs and found no effect (Fig. 5e). After long-term differentiation, M2 macrophages showed reduced Aβ-uptake capacity in both APOEε4( +) and APOEε4(-) -derived cells. However, after long-term compared to short-term M1 differentiation, we observed a difference between APOEε4(-) and APOEε4( +) in Aβ-uptake capacity, with decreased Aβ-uptake over time in APOEε4(-)-derived cells only (Fig. 5e, p_APOEε4(+)-M2shortvslong_ = 0.0144, p_APOEε4(-)-M2shortvslong_ = 0.0010 in M2 macrophages; p_APOEε4(-) M1shortvslong_ = 0.0031 in M1 macrophages). In APOEε4( +)-derived M1 and M2 macrophages, we observed a lower rate of Aβ-uptake after long-term vs short-term differentiation relative to APOEε4(-)-derived macrophages (Fig. 5f, U_M1_ = 38; p_M1_ = 0.051; U_M2_ = 28; p_M2_ = 0.01).

## Discussion

This is the first study to comprehensively explore the immunosenescence-related in vitro modulation of TREM2 and APOE in personalized Mo-MФ cultures in the presence of patient-specific circulating factors (AS), and the Aβ-uptake. Mo-MФs were differentiated for short-term (1 day) to long-term (10 days) from LOAD patients and CO. We show that TREM2 and APOE synthesis can be increased in M0- and M2- macrophages in AD-derived cells at levels comparable to those in CO-derived cells in a time-dependent manner. In this context, we report sex differences in the fold increase in TREM2 and APOE synthesis in AD-derived cells after long-term differentiation. We further point out the immunomodulatory role of human AS in APOE synthesis. Moreover, we demonstrated a time-dependent reduction in the Aβ-uptake capacity of M2-macrophages from both groups, while M1-macrophages showed reduced Aβ-uptake only in AD-derived cells. Finally, we report lower Aβ-uptake in APOEε4(+) compared to APOEε4(-) -derived cells.

Differentiation of monocytes into distinct subtypes of macrophages is strongly shaped by the tissue environment [55]. To better reflect their in vivo environment, we differentiated monocytes from CO and LOAD patients into M1- and M2- macrophages or left unstimulated (M0-macrophages) in the presence of the donors´ own sera [31]. Our results revealed comparable differentiation profiles and morphological changes of AD- and CO-derived cells in AS, as previously reported [24, 31]. These results support our previous findings, indicating that our in vitro assay accurately reflects differences in the inflammatory profiles of Mo-MФs not only in FCS but also in AS. Although several studies have indicated that elderly AD patients exhibit differential regulation of inflammatory processes triggered by age-associated decline in the immune system [38, 60], our results are consistent with other studies that did not detect such differences [40]. The immunoregulatory effect of human AS on peripheral blood cells in vitro has recently been shown for obese patients before and after exercise [56]. Our results, consistent with a previous report by Safi and colleagues [61], show that the AS condition significantly increases the yield of M0- macrophages with higher inflammatory marker secretion than the FCS condition.

TREM2 has been associated with several crucial innate immune functions including energy metabolism, survival and phagocytosis in AD pathology [62, 63]. The literature on *TREM2* levels in the central and peripheral compartments contains conflicting results, with implications for the influence of genetic variants, physiological conditions, and disease state [64–66]. In contrast to our previous results showing increased *TREM2* mRNA expression in CO- compared to AD-derived M2-macrophages in FCS [24], there was no difference in *TREM2* mRNA levels between AD- and CO-derived cells in our current results. This discrepancy between our current and previous results [24] could be explained by variability between study populations. In accordance with our previous work, however, our findings confirm that *TREM2* expression is increased in long-term M0- and M2- macrophages in AS condition as well. These findings complete previous reports showing an increased TREM2 expression only in M2-differentiated tissue macrophages [29, 67].

Changes in the levels of the proteolytic cleavage product of the cell surface TREM2 (sTREM2) have been proposed as a marker for monitoring AD progression [68]. The contradictory results so far in the published findings of sTREM2 levels in AD might likely to be due to differences in the stage of disease progression among the participants included in the studies [22, 69, 70]. While microglia are known to be the main source of sTREM2 in the CNS [23, 71], the degree to which other cells, such as brain-infiltrated macrophages, contribute to the levels of sTREM2 in CSF and peripheral blood during both normal and pathological aging in AD is unknown. Our data show that even in vitro “senescence” (10 days of differentiation, altogether 15 days of culture) increases the levels of sTREM2 in cell culture supernatants of Mo-MФs derived from both AD- and CO-derived cells. Notably, the reduced modulation of sTREM2 levels upon short-term differentiation of M0- and M2- macrophages in AD-derived cells was found to increase after long-term differentiation in AS, in agreement with our previous investigation in FCS [24]. Together, these results suggest that M0- and M2- macrophages from AD-derived cells together with microglia can be a main source of sTREM2 in the CNS.

While the primary source of brain APOE is astrocytes and microglia [72], a peripheral APOE pool has also been implicated in possibly affecting AD pathology [73, 74]. Previous findings suggest that APOE can modulate both its synthesis and anti-inflammatory macrophage function by promoting alternative M2 differentiation [30, 75]. Our data extend this finding by showing increased APOE secretion not only in M2- but also in M0-macrophages derived from CO- and AD-derived cells following long-term differentiation in the FCS condition. Our findings further suggest that the presence of circulatory factors present in human AS affects APOE synthesis after long-term differentiation in AS. Based on previous reports, it can be speculated that the regulatory effect of circulating factors such as lipogenic factors and/or cytokines in AS [75, 76] may cause this effect by differentially modulating the efficacy of M1 and M2 macrophages for APOE synthesis. With respect to potential disease contribution, a leaky blood–brain barrier that was shown in AD [77] may facilitate such factors to reach brain tissue to a larger extent in AD.

There is growing evidence that Mo-MФs accumulate in the brain during aging [9, 10] and that M2 macrophages constitute a small population that is thought to be further reduced in AD [29, 60]. Specifically, a reduction in these restorative cell populations (M2) [78], proposed to be the source of TREM2 and APOE, may potentiate disease progression [26, 27, 30]. Accordingly, it can be hypothesized that the continuous production of pro-inflammatory mediators and suppression of anti-inflammatory cytokines reduce the switch of infiltrating macrophages to the anti-inflammatory phenotype during disease progression [79]. While mouse models offer a wider range of experimental opportunities compared to human models, they may not accurately reflect age-related processes in human physiology due to the biological complexity of real-life patients with sporadic LOAD [80, 81]. Therefore, we suggest that in vitro senescence of macrophages with prolonged differentiation (e.g., 10 days) might be considered as a future characterization experiment of patient-derived cells that may offer a therapeutic approach through phenotypic transmission.

In addition to aging, sex-associated differences in the immune response are another risk factor for AD development [82]. Similar to monocytes [83], infiltrating Mo-MФs have also been shown to be sexually dimorphic in both phenotype and function [84]. Recent studies have demonstrated sex differences of infiltrating macrophages in controlling TREM2-dependent lipid homeostasis in obesity [85, 86]. Furthermore, it has been suggested that disruption of the homeostasis of lipid metabolism both in the CNS and in the periphery triggers the pathogenesis of AD [87]. Therefore, understanding the regulation of the TREM2 pathway in Mo-MФs in the context of aging and sex differences may better explain both AD-related comorbidities and how best to target the pathology in males and females. Our results indicate higher fold change in *TREM2* mRNA in female- AD-derived cells than in male- AD-derived cells after long-term M2 differentiation, while sTREM2 levels are relatively lower. Our findings also indicate significantly lower levels of APOE in female vs. male AD-derived macrophages. We believe that a better understanding of the biology of TREM2-expressing macrophages during aging may offer insights into the molecular underpinnings of sex differences in AD.

Growing evidence suggests that aging is associated with relative loss of function in microglia [88, 89]. Thus, some reports suggest that bone marrow-derived cells might play an important role in preventing disease progression in the CNS, by replenishing a failing microglial system [12, 90, 91]. Notably, infiltrating monocytes have been reported to account for 6% of plaque-associated macrophages in aged AD mice [12] and show significant activity in a microglia-like phenotype during phagocytosis [13]. However, there is a gap in understanding the effect of aging on the Aβ-uptake ability of Mo-MФs at the patient level. Our findings show decreased Aβ-uptake in in vitro senescent M2 macrophages after long-term differentiation from both groups. Furthermore, M1 macrophages exhibit markedly reduced Aβ-uptake in AD-derived cells only. In this context, the literature on the Aβ-uptake ability of Mo-MФs is contradictory [12, 92], possibly due to different experimental methods or phagocytes altering the monocyte phenotype and response. Our results suggest that the reduced Aβ uptake ability of M1 and M2 macrophages after long-term differentiation resembles that of senescent microglia. Nevertheless, the functional effectiveness of both senescent Mo-MФs and microglia remains to be elucidated.

The APOEε4 variant is the most common genetic risk factor for AD and is associated with dysregulation of cellular functions in AD pathology [93]. Microglia-like cells derived from APOEε4(+) iPSCs were shown to exhibit slower Aβ_42_ uptake in AD compared to those derived from APOEε3(+) [94]. In line with previous reports, our results suggest that cells derived from APOEε4(+) carriers show a decrease in their Aβ-uptake capacity, as characterized by a fold decrease in M1- and M2-macrophages after long-term compared to short-term differentiation. [94, 95]. Thus, investigating the mechanism driving the APOEε4-mediated immune response may help understand how intrinsic dysregulation of APOEε4 affects selective phagocytosis and disease progression.

Our study aimed to investigate for the first time how TREM2 and APOE levels, together with changes in Aβ-uptake, are modulated in an in vitro model of patient-specific senescent Mo-MФs, considering sex and APOEε4 genotype. Accurately representing the molecular and cellular features of the disease at the patient-specific level is the strength of our study, making it a valuable tool to investigate disease mechanisms and develop treatment strategies [24, 31]. However, our current study has some limitations that should be considered when interpreting the findings. First, we did not screen for possible TREM2 mutations that might affect expression or function, but this is unlikely to have affected our results, as TREM2 mutations are rare both in the general population and in AD [96–98]. Second, while we have evidence from previous reports for the role of the TREM2-APOE pathway in Aβ-uptake [18, 57] our current results cannot address this.

## Conclusion

The present study is the first to assess the dynamics of TREM2 and APOE in a patient-specific Mo-MФ differentiation assay under physiologically relevant conditions (AS). We report increased TREM2 and APOE protein levels in AD- and CO-derived cells in a time-dependent manner, which was more pronounced in M2 macrophages. Furthermore, we report sex differences in TREM2 and APOE synthesis in AD-derived cells only, noting that cells derived from females showed a higher fold increase in *TREM2*, but a lower fold increase in APOE secretion compared to males following long-term differentiation. Moreover, we show reduced Aβ-uptake in APOEε4(+)-derived cells. Given the neuroprotective properties attributed to M2 macrophages, it is plausible that a shift in the macrophage phenotype towards an M2 state may lead to a substantial boost in TREM2 and APOE synthesis in AD patients. Hence, promoting a shift in macrophage phenotype towards M2 could be a promising therapeutic strategy in AD. In accordance, the use of an appropriate stimulation duration in in vitro personalized cell culture experiments may provide a potential opportunity for precision medicine-based development or screening of novel therapeutic approaches for AD.

### Supplementary Information

**Additional file 1: Supplemental Table 1.**  Cytokines used for differentiation. **Supplemental Table 2.  **Detection range of measured cytokines (pg/ml). **Supplementary Table 3.**  Modulation of neuroinflammatory marker synthesis in short- and long-term differentiated Mo-MФs in autologous serum (AS). **Supplementary Table 4. **Serum effect on short- and long-term M0, M1 and M2 macrophage differentiation. **Supplementary Table 5.  **Serum effect on TREM2 and APOE synthesis in short- and long-term patient-derived Mo-MФs cultures. **Supplementary Table 6.** Time effect on TREM2 and APOE synthesis in short- and long-term Mo-MФs cultures in autologous serum (AS). **Supplementary Table 7. **Sex differences in TREM2 and APOE levels (fold changes) in Mo-MФs cultures in autologous serum (AS). 

**Additional file 2: Supplemental Fig. 1.** FCS modulation of TREM2 in patient-specific Mo-MФs, and sex and genotype effect in AS. (a) *TREM2* mRNA and (b) sTREM2 synthesis in Mo-MФ cultures from AD patients (*n*=21) and CO (*n*=16) in FCS. Effect of sex on (c) *TREM2* mRNA and (d) sTREM2 levels in Mo-MФs from female -AD (*n*=10) vs. -CO (*n*=11) and male -AD (*n*=11) vs -CO (*n*=5) derived cells in AS. Genotype effect on (e) *TREM2* mRNA and (f) sTREM2 levels in Mo-MФs from APOEε4(+)-AD (*n*=10) vs. APOEε4(+)-CO (*n*=6) and APOEε4(-)-AD (*n*=11) vs APOEε4(-)-CO (*n*=10) derived cells in AS. (a-b) Closed bars and symbols represent M0 (light grey for short-term; dark grey for long-term), M1 (light blue for short-term; dark blue for long-term) and M2 (light orange for short-term; dark orange for long-term) macrophages respectively (c-f) Closed bars and symbols represent CO (light grey for short-term; dark grey for long-term) and AD (light red for short-term; dark red for long-term). Dots represent individual participant values. mRNA (normalized to GAPDH) expression was measured with RT-qPCR. The Friedman ANOVA was used to compare within-group differences, while the Kruskal-Wallis test (paired groups) was used to assess between-group differences (p*<0.0167 (p/n, assuming n = 3 comparison). **Supplemental Fig. 2.** Sex or APOEε4 genotype does not modulate APOE synthesis in short- and long-term Mo-MФs. Effect of sex on APOE secretion levels in (a) FCS and (b) AS supplemented Mo-MФs cultures from female-AD (*n*=10) vs. -CO (*n*=11) and male-AD (*n*=11) vs –CO (*n*=5). Genotype effect on APOE secretion levels in (c) FCS and (d) AS supplemented Mo-MФ cultures from APOEε4(+)-AD (*n*=10) vs. -CO (*n*=6) and APOE ε4 (-)-AD (*n*=11) vs –CO (*n*=10). (a-d) Closed bars and symbols represent CO (light grey for short-term; dark grey for long-term) and AD (light red for short-term; dark red for long-term). Dots represent individual participant values. mRNA (normalized to GAPDH) expression was measured with RT-qPCR.  The group differences were analysed by the Kruskal Wallis test for pairwise comparisons followed by Dunn’s multiple comparisons test with Bonferroni correction (p*<0.0167 (p/n, assuming n = 3 comparison). **Supplementary Fig. 3.** Sex did not modulate relative TREM2 and APOE levels. Fold increase in (a) *TREM2* mRNA, (b) sTREM2 and (c) APOE levels in Mo-MФ cultures from CO-female patients (*n*=11) vs. AD-female patients (*n*=10) after long-term compared to short-term differentiation in AS. Fold increase in (d) *TREM2* mRNA, (e) sTREM2 and (f) APOE levels in Mo-MФs cultures from CO-male patients (*n*=5) vs. AD-male patients (*n*=11) after long-term compared to short-term differentiation in AS. (a-f) Closed bars and symbols represent CO (light grey for females; dark grey males) vs. AD (red for females; blue for males). mRNA (normalized to GAPDH) expression was measured with RT-qPCR. A pairwise comparison of groups was performed with the Mann Whitney U-test (paired groups) (*p<0.05, **p<0.01,***p<0.001). **Supplementary Fig. 4.** Sex or APOEε4 genotype does not modulate Aβ-uptake in M1- and M2- macrophages within groups. Sex differences in Aβ-uptake levels in (a) AD (female=6 vs. male=6) and (b) CO- (female=4 vs. male=8) derived M1- and M2- macrophages. Genotype effect on Aβ-uptake levels in (a) AD (APOEε4(+)=6 vs. APOEε4(-)=6) and (b) CO- (APOEε4(+)=6 vs. APOEε4(-)=6) derived M1- and M2- macrophages. (a-d) Closed bars and symbols represent female (red) vs. male (blue) and APOEε4(+) (purple) vs. APOEε4(-) (green) derived cells within groups. Dots represent individual participant values. The group differences were analysed by the Kruskal Wallis test for pairwise comparisons followed by Dunn’s multiple comparisons test with Bonferroni correction (p*<0.0167 (p/n, assuming *n* = 3 comparison).

## Data Availability

All relevant data in this study are available upon reasonable request directed to the corresponding author.

## Abbreviations

LOAD: Late-onset Alzheimer´s disease
Mo-MФs: Monocyte-derived macrophages
CNS: Central nervous system
AD: Alzheimer`s disease
TREM2: Triggering receptor expressed on myeloid cells 2
APOE: Apolipoprotein E
Aβ: Amyloid beta
CO: Control
LPS: Lipopolysaccharide
IL-4: Interleukin-4
IL-10: Interleukin-10
TGF-β: Transforming growth factor beta
IL-13: Interleukin-10
sTREM2: Soluble triggering receptor expressed on myeloid cells 2
CSF: Cerebrospinal fluid
BBB: Blood–brain barrier
M-CSF: Macrophage-colony stimulating factor
DAMPs: Damage-associated molecular patterns
CRP: C-reactive protein
IL-6: Interleukin-6
TNF- α: Tumor necrosis factor alpha
FCS: Fetal calf serum
BMI: Body-mass index
NIA: National Institute on Aging
PBMCs: Peripheral blood mononuclear cells
RT: Room temperature
P/S: Penicillin/Streptomycin
BCA: Bicinchoninic acid
MCP-1: Monocyte chemoattractant protein-1
CV: Coefficient variant
PBS: Phosphate‐buffered saline
PFA: Paraformaldehyde
IQR: Inter-quartile range
ANOVA: Analysis of variance
MFI: Mean fluorescence intensity
MMSE: Mini mental state examination
T-tau: Total tau
P-tau: Phosphorylated tau
LAM: Lipid-associated macrophages
DAM: Disease-associated macrophages
GAPDH: Glyceraldehyde-3-phosphate dehydrogenase
ΔCt: Data normalization relative to housekeeping gene
