# Supplementary material for: Effects of autologous serum on TREM2 and APOE in a personalized monocyte-derived macrophage assay of late-onset Alzheimer’s patients
Source: Immun Ageing. 2023 Oct 14;20:52. doi: 10.1186/s12979-023-00376-2 (PMC10576307; doi:10.1186/s12979-023-00376-2)
Supplement: Supplementary file 1 — Additional file 1: Supplemental Table 1. Cytokines used for differentiation. Supplemental Table 2. Detection range of measured cytokines (pg/ml). Supplementary Table 3. Modulation of neuroinflammatory marker synthesis in short- and long-term differentiated Mo-MФs in autologous serum (AS). Supplementary Table 4. Serum effect on short- and long-term M0, M1 and M2 macrophage differentiation. Supplementary Table 5. Serum effect on TREM2 and APOE synthesis in short- and long-term patient-derived Mo-MФs cultures. Supplementary Table 6. Time effect on TREM2 and APOE synthesis in short- and long-term Mo-MФs cultures in autologous serum (AS). Supplementary Table 7. Sex differences in TREM2 and APOE levels (fold changes) in Mo-MФs cultures in autologous serum (AS). [file 12979_2023_376_MOESM1_ESM.docx]

| Supplemental Table 1. Cytokines used for differentiation | | |
| --- | --- | --- |
| source | **Type of Differentiation** | **Differentiation Agents** |
| *Human* | M1- Macrophage | 50 ng/ml LPS; L6529 |
|  | M2-Macrophage | 20 ng/ml IL-4; Lot 091514  20 ng/ml IL-10; Lot 110621  20 ng/ml TGF-β ; Lot 0506S354 |
|  | M0- Macrophage | 10 ng/ml M-CSF |
| *LPS- Lipopolysaccharide ; IL-4-Interleukin-4; IL-10-Interleukin-10 ; TGF-ß -Transforming growth factor beta; M-CSF-Macrophage colony-stimulating factor* | | |

| **Supplemental Table 2.** Detection range of measured cytokines (pg/ml) | | |
| --- | --- | --- |
|  | **Short-term Differentiation** | **Long-term Differentiation** |
| sTREM2 | 3.010 - 1929.01 | 3.010 - 13737.36 |
| APOE | 526.97 -892857.59 | 2851.87 -1731787.86 |
| TGF-β | 3.204 - 18026.69 | 3.204 - 14557.81 |
| IL-6 | 3.16 - 8939.857 | 3.16 - 9905.366 |
| TNF-α | 2.895-3783.89 | 2.895-253.68 |
| MCP-1 | 3.43 - 3784.4 | 3.43 - 4871.58 |
| *sTREM2*- soluble triggering receptor expressed on myeloid cells 2; APOE- apolipoprotein E. IL-6 -interleukin 6; TNFα- tumor necrosis factor α; MCP-1 - monocyte chemoattractant protein-1; TGF-β -transforming growth factor beta β | | |

| Supplementary Table 3. Modulation of neuroinflammatory marker synthesis in short- and long-term differentiated Mo-MФs in autologous serum (AS). | | | | | | | | | | | | | | | |
| --- | --- | --- | --- | --- | --- | --- | --- | --- | --- | --- | --- | --- | --- | --- | --- |
|  | **Short-term** | | | | | **Long-term** | | | | | | | | | |
| CO (n=16) | | | | | | | | | | | | | | | |
| Protein | Friedman statistic | p-value | Dunn´s multiple comparison tests | | | Friedman statistic | | p-value | | Dunn´s multiple comparison tests | | | | | |
|  |  |  | M1 vs. M2 | M1 vs. M0 | M2 vs. M0 | |  |  | | M1 vs. M2 | | M1 vs. M0 | | M2 vs. M0 | |
| IL-6 | 21.24 | <0.0001 | **0.0106** | **<0.0001** | 0.3348 | | 21.13 | <0.0001 | | **0.0001** | | **0.0003** | | >0.9999 | |
| TNF- α | 23.46 | <0.0001 | **0.0032** | **<0.0001** | 0.4719 | | 14.51 | 0.0007 | | **0.0009** | | 0.0184 | | >0.9999 | |
| TGF-β | 30.13 | <0.0001 | **<0.0001** | 0.040 | **0.008** | | 32 | <0.0001 | | **<0.0001** | | **0.0140** | | **0.0140** | |
| mcp-1 | 7.625 | 0.0221 | 0.0647 | 0.040 | >0.9999 | | 5.375 | 0.0681 | | >0.9999 | | 0.4719 | | 0.0647 | |
| AD (n=21) | | | | | | | | | | | | | | | |
| IL-6 | 29.81 | <0.0001 | **0.0021** | **<0.0001** | 0.1346 | | 23.52 | | <0.0001 | | **<0.0001** | | **0.0061** | | 0.2689 |
| TNF- α | 29.81 | <0.0001 | **0.0021** | **<0.0001** | 0.1346 | | 4.072 | | 0.1305 | | 0.2279 | | 0.2689 | | >0.9999 |
| TGF-β | 42 | <0.0001 | **<0.0001** | **0.0036** | **0.0036** | | 42 | | <0.0001 | | **<0.0001** | | **0.0036** | | **0.0036** |
| mcp-1 | 12.10 | 0.0024 | **0.0061** | >0.9999 | **0.0101** | | 4.095 | | 0.1290 | | 0.6511 | | >0.9999 | | 0.1346 |
| Protein levels calculated using Friedman ANOVA Test with significance values adjusted by the Bonferroni corrections (p<0.0167) for Dunn´s multiple comparison tests for all measured markers in M1. M2. and M0 after short- and long-term differentiation. IL-6 -interleukin 6; TNFα- tumor necrosis factor α; MCP-1 - monocyte chemoattractant protein-1; TGF-β -transforming growth factor beta β;. | | | | | | | | | | | | | | | |

| **Supplementary Table 4.** Serum effect on short- and long-term M0, M1 and M2 macrophage differentiation | | | | | | | | |
| --- | --- | --- | --- | --- | --- | --- | --- | --- |
|  | **Short-term differentiation** | | | | | | | |
|  | **CO (n=16)** | | | | **AD (n=21)** | | | |
| **Type of Differentiation** | **FCS** Median (25% - 75%) | **AS**  Median (25% - 75%) | Wilcoxon test | p-value | **FCS**  Median (25% - 75%) | **AS**  Median (25% - 75%) | Wilcoxon test | p-value |
| M0 | -2.750 (-3.6 - -2.05) | -1.03 (-1.696- 0.859) | 134 | **<0.0001** | -3.1 (-4 - -2.4) | 0.2 (-1.2- 1.05) | 231 | **<0.0001** |
| M1 | 2 (-0.22 – 3.584) | 0.784 (-0.172- 4.74) | 22 | 0.5966 | 1.987 (0.204-3.026) | 0.179 (-0.213- 1.71) | -119 | **0.0384** |
| M2 | 1.77 (0.9825 – 1.94) | 1.13 (0.97 – 1.455) | -62 | 0.0806 | 1.05 (0.885- 1.785) | 1.11 (0.955-1.340) | -66 | 0.2609 |
|  | **Long-term differentiation** | | | | | | | |
|  | **CO (n=16)** | | | | **AD (n=21)** | | | |
| **Type of Differentiation** | **FCS** Median (25% - 75%) | **AS**  Median (25% - 75%) | Wilcoxon test | p-value | **FCS**  Median (25% - 75%) | **AS**  Median (25% - 75%) | Wilcoxon test | p-value |
| M0 | -1.696 (-2.26 - -0.03) | 0.439 (-1.145- 2.28) | 110 | **0.0027** | -1.6 (-2.83 – 0.94) | 0.4 (-1.12- 2.6) | 167 | **0.0025** |
| M1 | 0.622 (-0.499-3.602) | 0.753 (-0.334-1.949) | -28 | 0.4954 | 0.376 (-0.329-1.5) | -0.015 (-0.38-0.667) | -79 | 0.179 |
| M2 | 1.99 (0.877- 2.128) | 0.79 (0.4775-1.055) | -109 | **0.0029** | 2.02 (1.17-2.185) | 0.77 (0.485-1.055) | -195 | **0.0002** |
| *Serum effect* calculated using Wilcoxon signed-rank test **(p<0.05).** | | | | | | | | |

| **Supplementary Table 5.** Serum effect on TREM2 and APOE synthesis in short- and long-term patient-derived Mo-MФs cultures. | | | | | | | | |
| --- | --- | --- | --- | --- | --- | --- | --- | --- |
|  | **Short-term M1 macrophages** | | | | | | | |
|  | **CO (n=16)** | | | | **AD (n=21)** | | | |
| ***Gene**/**  **Protein** | **FCS** Median (25% - 75%) | **AS**  Median (25% - 75%) | Wilcoxon test | p-value | **FCS**  Median (25% - 75%) | **AS**  Median (25% - 75%) | Wilcoxon test | p-value |
| *TREM2* | 0.224 (0.05 – 1.816) | 0.19 (0.0.97 – 0.436) | -50 | 0.2114 | 0.261 (0.116 – 3.11) | 0.233 (0.138-0.319) | -93 | 0.1111 |
| sTREM2 | 0.574 (0.266 – 0.96) | 0.749 (0.53-0.929) | 12 | 0.782 | 0.936 (0.544 – 1) | 0.792 (0.417 – 0.969) | -69 | 0.2428 |
| APOE | 1.012 (0.205 – 2.644) | 1.08 (0.93 – 1.183) | -26 | 0.528 | 0.971 (0.589 – 2.20) | 0.9705 (0.77-1.293) | -60 | 0.2428 |
|  | **Short-term M2 macrophages** | | | | | | | |
|  | **CO (n=16)** | | | | **AD (n=21)** | | | |
| ***Gene**/**  **Protein** | **FCS** Median (25% - 75%) | **AS**  Median (25% - 75%) | Wilcoxon test | p-value | **FCS**  Median (25% - 75%) | **AS**  Median (25% - 75%) | Wilcoxon test | p-value |
| *TREM2* | 1.498 (0.995 – 2.105) | 0.951 (0.434 – 1.51) | -46 | 0.2522 | 1.406 (0.268-2.28) | 1.254 (0.886 – 1.646) | -13 | 0.8382 |
| sTREM2 | 1.176 (0.759 – 1.403) | 0.965 (0.641 – 1.12) | -16 | 0.7057 | 0.955(0.435 – 1.109) | 0.965 (0.676 – 1.371) | 13 | 0.8382 |
| APOE | 1.04 ( 0.369 – 2.247) | 0.941(0.816 – 1.188) | -44 | 0.2744 | 1.444 (0.7 – 4.439) | 1.026 (0.698 – 1.217) | -131 | **0.0216** |
|  | **Long-term M1 macrophages** | | | | | | | |
|  | **CO (n=16)** | | | | **AD (n=21)** | | | |
| ***Gene**/**  **Protein** | **FCS** Median (25% - 75%) | **AS**  Median (25% - 75%) | Wilcoxon test | p-value | **FCS**  Median (25% - 75%) | **AS**  Median (25% - 75%) | Wilcoxon test | p-value |
| *TREM2* | 0.678 (0.257 – 3.32) | 0.705 (0.41 – 0.945) | -38 | 0.348 | 0.33 (0.216 - 0.657) | 0.43 (0.302 - 0.779) | 87 | 0.1373 |
| sTREM2 | 0.17(0.094 – 0.698) | 0.275 (0.2 – 0.7) | 54 | 0.1754 | 0.148(0.045 – 0.552) | 0.273 (0.17 - 0.803) | 81 | 0.1678 |
| APOE | 0.153 (0.058 – 0.549) | 0.94 (0.584 – 1.134) | 124 | **0.0004** | 0.33 (0.127 – 0.772) | 1.05 (0.751 – 1.233) | 229 | **<0.0001** |
|  | **Long-term M2 macrophages** | | | | | | | |
|  | **CO (n=16)** | | | | **AD (n=21)** | | | |
| ***Gene**/**  **Protein** | **FCS** Median (25% - 75%) | **AS**  Median (25% - 75%) | Wilcoxon test | p-value | **FCS**  Median (25% - 75%) | **AS**  Median (25% - 75%) | Wilcoxon test | p-value |
| *TREM2* | 2.813 (2.03-3.18) | 2.56 (1.49-4.42) | 14 | 0.744 | 1.71 (1.405 – 1.89) | 1.693(0.89 – 2.617) | 3 | 0.9729 |
| sTREM2 | 1.215 (0.58 – 2.197) | 0.861 (0.579 – 1.06) | -62 | 0.1167 | 1.758 (0.947-3.024) | 0.989(0.6521-1.331) | -133 | **0.0195** |
| APOE | 1.359 (0.840 – 3.166) | 0.889 (0.598-1.044) | -116 | **0.0013** | 1.545 (0.565 – 4.91) | 0.852 (0.693 – 1.072) | -171 | **0.0019** |
| **qPCR products in italic and p*roteins calculated using Wilcoxon signed-rank test. *TREM*2- triggering receptor expressed on myeloid cells 2; sTREM2- soluble triggering receptor expressed on myeloid cells 2; APOE- apolipoprotein E. | | | | | | | | |

| Supplementary Table 6. Time effect on TREM2 and APOE synthesis in short- and long-term Mo-MФs cultures in autologous serum (AS). | | | | | | | | | | |  |
| --- | --- | --- | --- | --- | --- | --- | --- | --- | --- | --- | --- |
| Gene*/  Protein | **Short-term vs. Long-term** | | | | | | | | | |  |
|  | **CO (n=16)** | | | | | **AD (n=21)** | | | | |  |
|  | Friedman statistic | p-value | Dunn´s multiple comparison tests | | | Friedman statistic | p-value | Dunn´s multiple comparison tests | | |  |
|  |  |  | M0 vs. M0 | M1 vs. M1 | M2 vs. M2 |  |  | M0 vs. M0 | M1 vs. M1 | M2 vs. M2 |  |
| *TREM2* | | 38.25 | <0.0001 | 0.1417 | >0.9999 | 0.4691 | 35.75 | <0.0001 | >0.9999 | 0.2971 | >0.9999 |
| sTREM2 | | 54.79 | <0.0001 | **<0.0001** | **0.0039** | **0.0003** | 55.88 | <0.0001 | **<0.0001** | **0.0022** | **<0.0001** |
| APOE | | 14.32 | 0.0137 | **0.0075** | 0.2669 | 0.3917 | 19.48 | 0.0016 | 0.0503 | 0.0400 | 0.0960 |
| *qPCR products* (in italic)* and proteins calculated using Friedman ANOVA Test with significance values adjusted by the Bonferroni corrections (p<0.0167) for Dunn´s multiple comparison tests for TREM2- triggering receptor expressed on myeloid cells 2; sTREM2- soluble triggering receptor expressed on myeloid cells 2; APOE- apolipoprotein E after short- and long-term differentiation | | | | | | | | | | |  |

| **Supplementary Table 7.** Sex differences in TREM2 and APOE levels (fold changes) in Mo-MФs cultures in autologous serum (AS). | | | | | | | | |
| --- | --- | --- | --- | --- | --- | --- | --- | --- |
|  | ***TREM2 mRNA*** | | | | | | | |
|  | **CO (n=16)** | | | | **AD (n=21)** | | | |
| **Type of Differentiation** | **Female** Median (25% - 75%) | **Male**  Median (25% - 75%) | Mann  Whitney U test | p-value | **Female** Median (25% - 75%) | **Male**  Median (25% - 75%) | Mann Whitney U test | p-value |
| M0 | 0.455 (0.167-1.282) | 0.163(0.081-1.747) | 24 | 0.743 | 1.09 (0.275 – 2.019) | 0.569(0.285-0.778) | 33 | 0.1273 |
| M1 | 1.129 (0.418-2.514) | 4.748(1.476-9.856) | 14 | 0.1451 | 2.81 (0.59-5.27) | 1.245 (0.89-4.88) | 55 | >0.999 |
| M2 | 2.057(0.532-2.878) | 1.111(0.314-1.908) | 17 | 0.2674 | 1.996 (0.782-3.2) | 0.486(0.275-0.933) | 21 | **0.0159** |
|  | **sTREM2** | | | | | | | |
|  | **CO (n=16)** | | | | **AD (n=21)** | | | |
| **Type of Differentiation** | **Female** Median (25% - 75%) | **Male**  Median (25% - 75%) | Mann Whitney U test | p-value | **Female** Median (25% - 75%) | **Male**  Median (25% - 75%) | Mann Whitney U test | p-value |
| M0 | 8.82 (2.98-98.93) | 14.29 (6.30-22.27) | 26 | 0.913 | 13.76 (1.54-32.68) | 32.67(8.54-62.6) | 40 | 0.3144 |
| M1 | 6.494(2.072-7.89) | 4.82 (2.47-7.166) | 25 | 0.8061 | 6.46 (2.92-14.87) | 8.212(1.464-16.49) | 55 | >0.999 |
| M2 | 10.17 (2.278-34.08) | 8.61(7.93-36.77) | 23 | 0.661 | 12.18(6.93-21.7) | 18.9(7.44-72.94) | 45 | 0.5116 |
|  | **APOE** | | | | | | | |
|  | **CO (n=16)** | | | | **AD (n=21)** | | | |
| **Type of Differentiation** | **Female** Median (25% - 75%) | **Male**  Median (25% - 75%) | Mann Whitney U test | p-value | **Female** Median (25% - 75%) | **Male**  Median (25% - 75%) | Mann Whitney U test | p-value |
| M0 | 1.422(1.136-1.869) | 1.754(1.049-3.81) | 23 | 0.6612 | 0.911 (0.625-1.72) | 2.187(1.529-3.014) | 17 | **0.0062** |
| M1 | 1.459(1.162-1.678) | 1.326(0.579-1.482) | 18 | 0.3198 | 1.039(0.941-1.187) | 1.665(1.146-2.144) | 25 | **0.0357** |
| M2 | 1.526(0.6741-1.815) | 1.115(0.9707-2.232) | 27 | >0.999 | 1.069(0.688-1.48) | 1.821(1.460-2.29) | 18 | **0.0079** |
| Sex differences in qPCR products* (in italic) and proteins calculated using Mann Whitney U test **(p<0.05).** TREM2- triggering receptor expressed on myeloid cells 2; sTREM2- soluble triggering receptor expressed on myeloid cells 2; APOE- apolipoprotein E. | | | | | | | | |
