# Supplementary figures and images for: Effects of autologous serum on TREM2 and APOE in a personalized monocyte-derived macrophage assay of late-onset Alzheimer’s patients
Source: Immun Ageing. 2023 Oct 14;20:52. doi: 10.1186/s12979-023-00376-2 (PMC10576307; doi:10.1186/s12979-023-00376-2)

## Slide 1
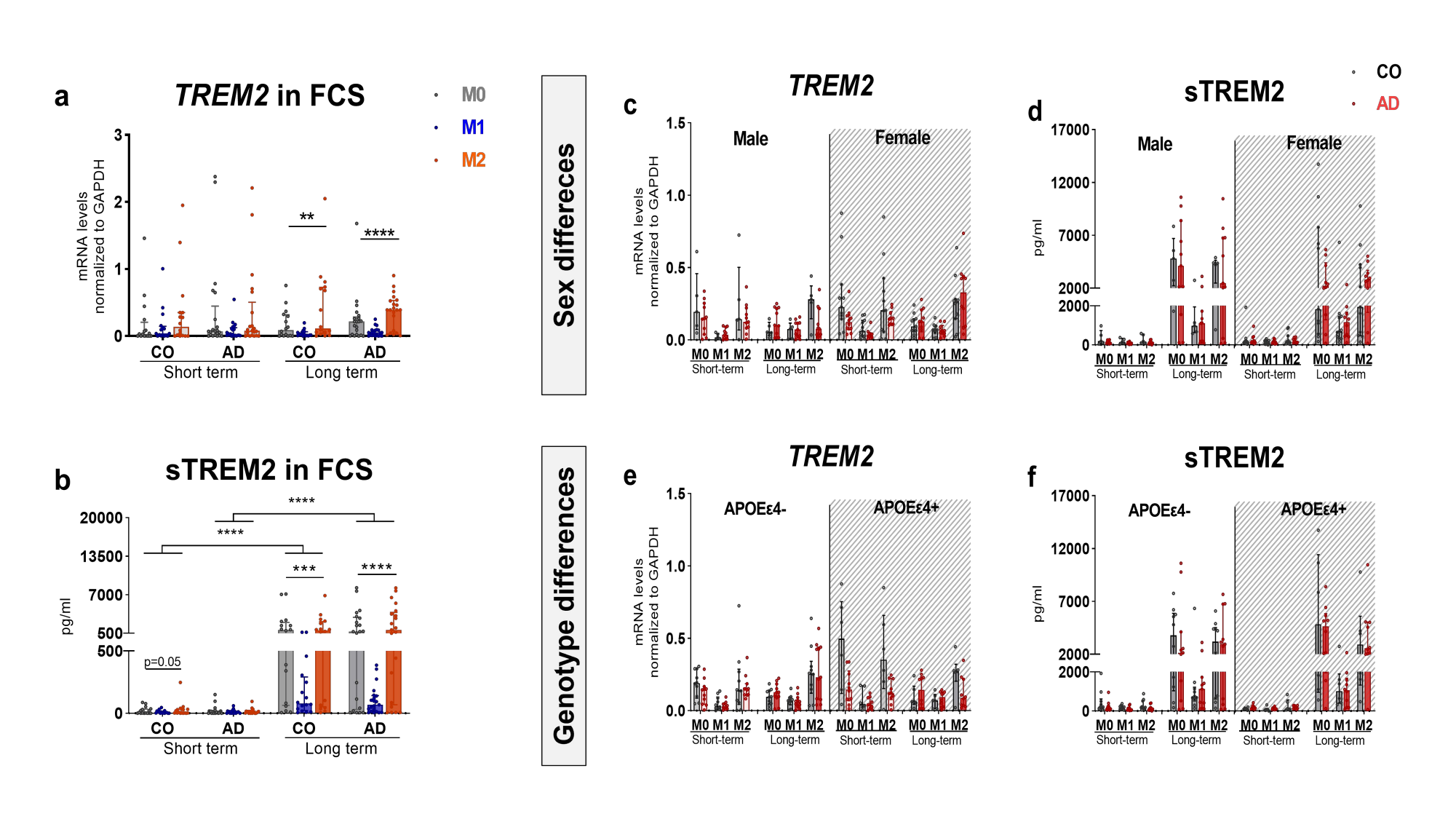

## Slide 2
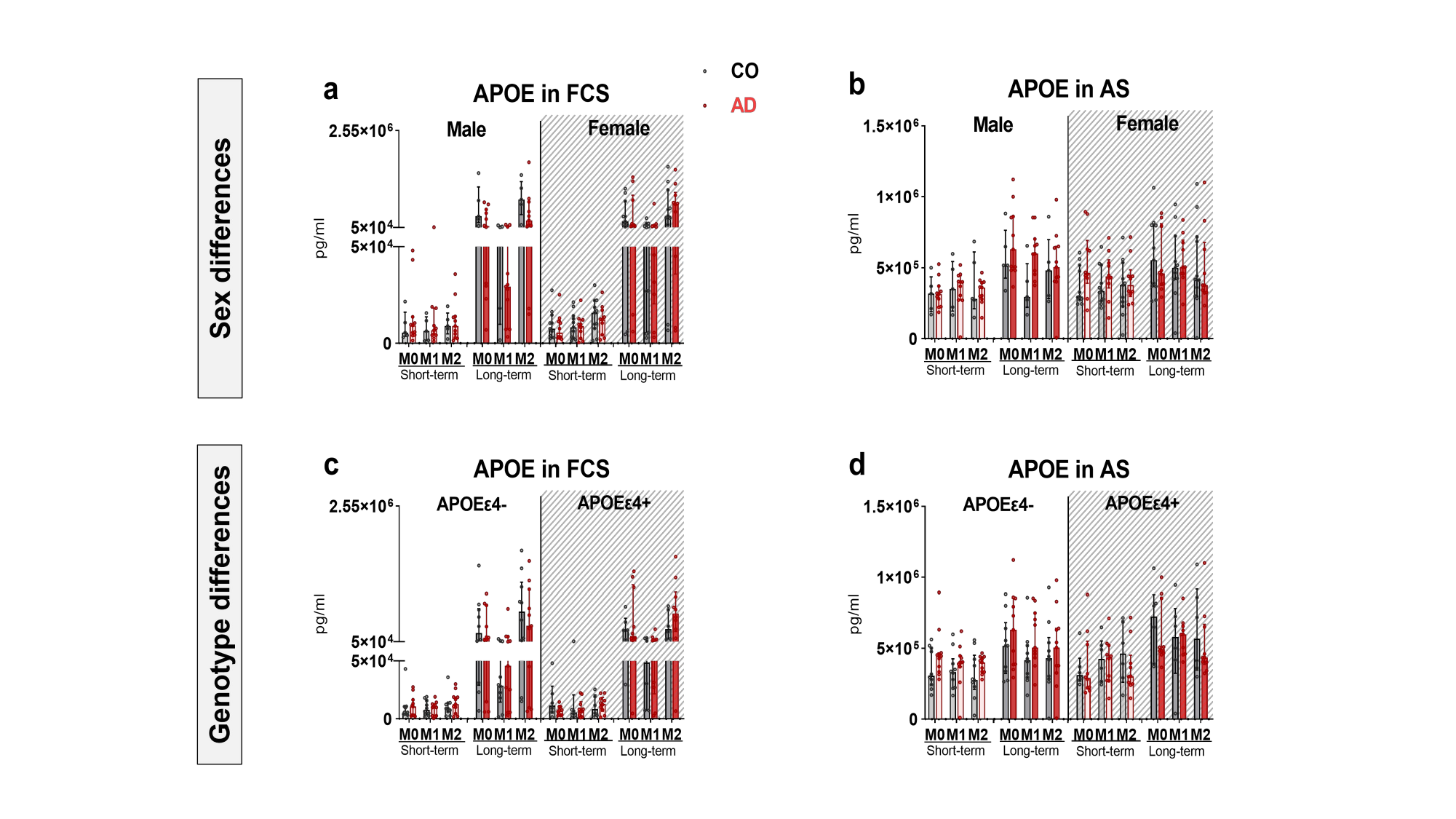

## Slide 3
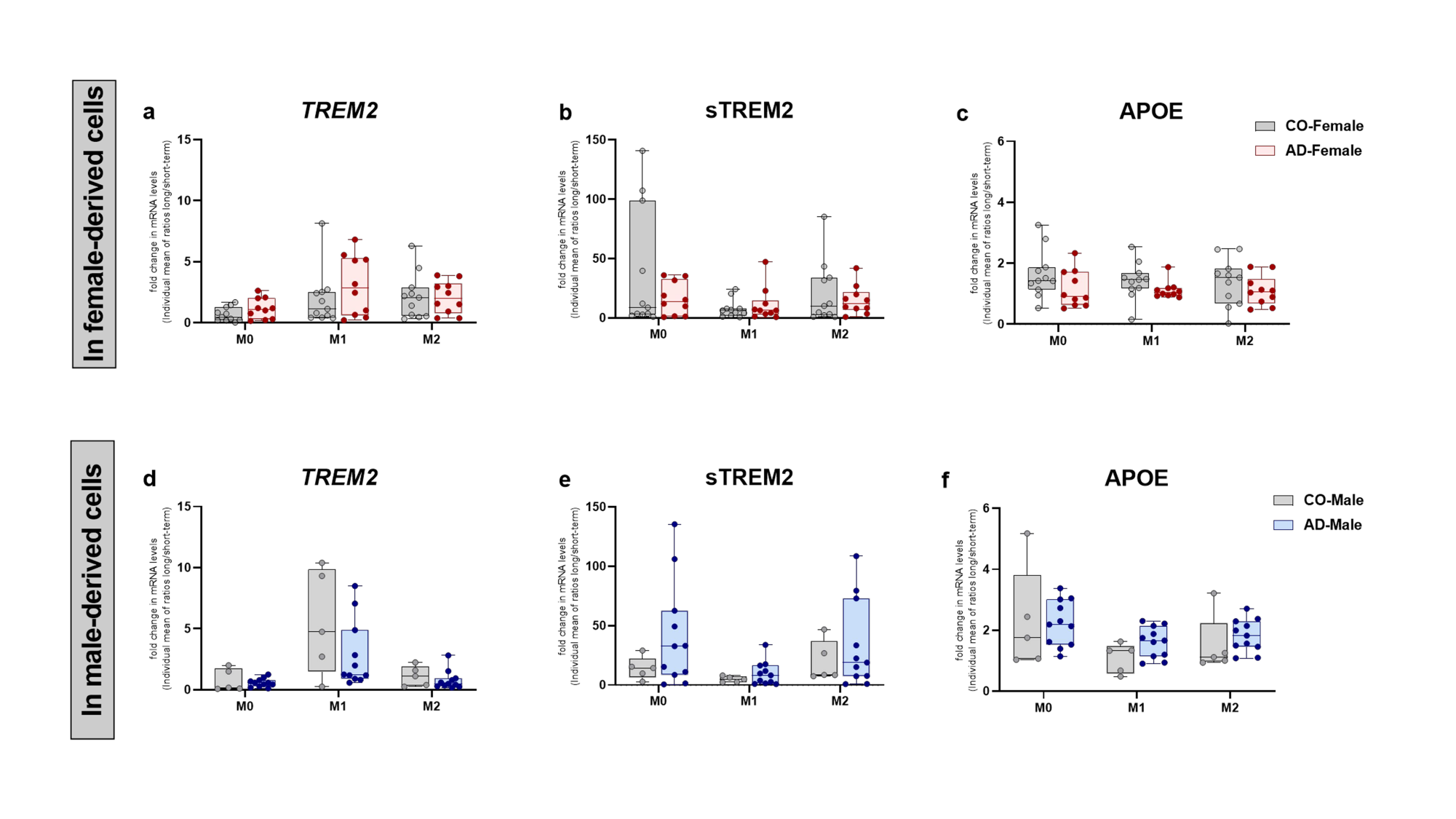

## Slide 4
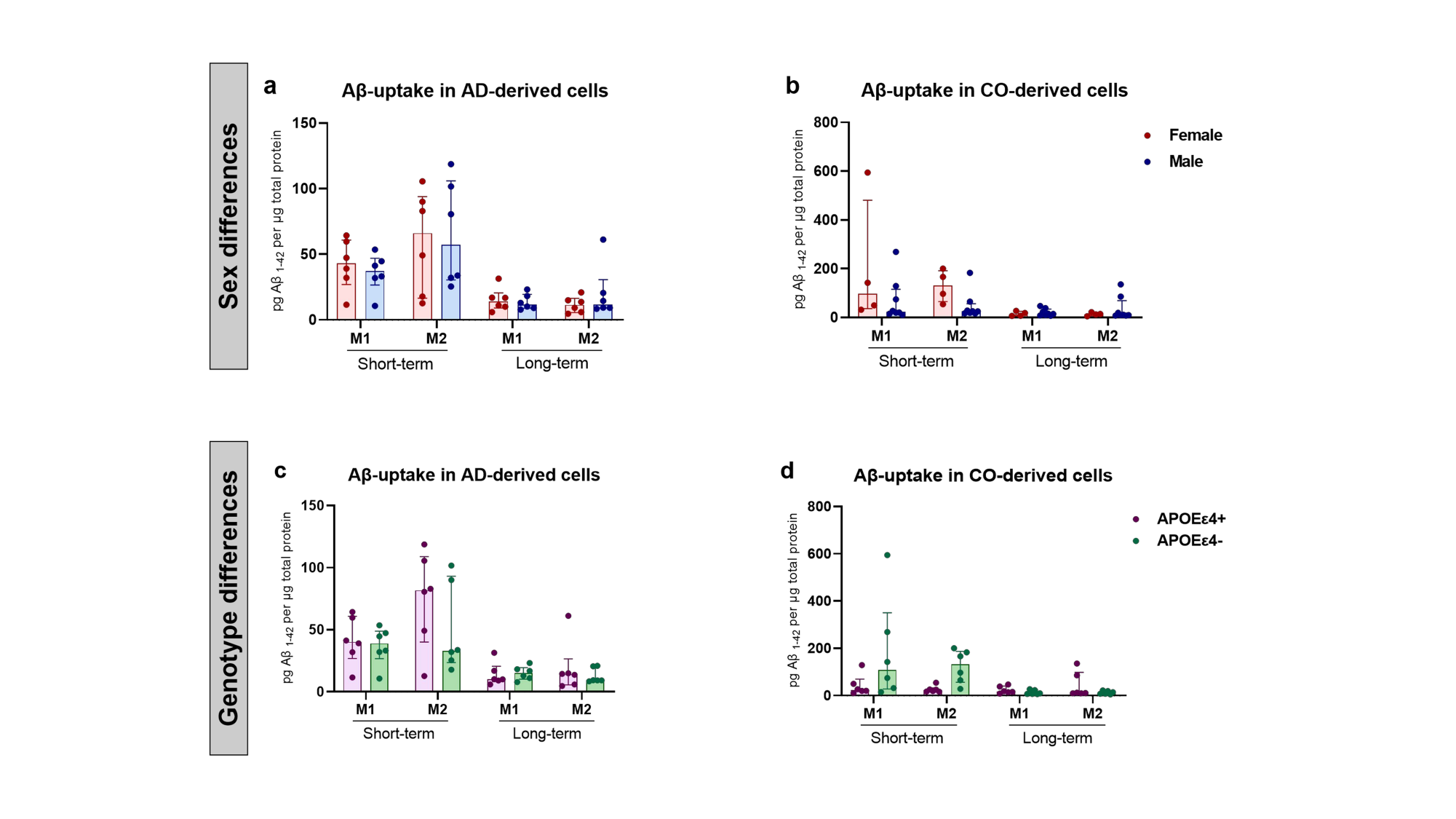

Supplement: Supplementary file 2 — Additional file 2: Supplemental Fig. 1. FCS modulation of TREM2 in patient-specific Mo-MФs, and sex and genotype effect in AS. (a) TREM2 mRNA and (b) sTREM2 synthesis in Mo-MФ cultures from AD patients (n=21) and CO (n=16) in FCS. Effect of sex on (c) TREM2 mRNA and (d) sTREM2 levels in Mo-MФs from female -AD (n=10) vs. -CO (n=11) and male -AD (n=11) vs -CO (n=5) derived cells in AS. Genotype effect on (e) TREM2 mRNA and (f) sTREM2 levels in Mo-MФs from APOEε4(+)-AD (n=10) vs. APOEε4(+)-CO (n=6) and APOEε4(-)-AD (n=11) vs APOEε4(-)-CO (n=10) derived cells in AS. (a-b) Closed bars and symbols represent M0 (light grey for short-term; dark grey for long-term), M1 (light blue for short-term; dark blue for long-term) and M2 (light orange for short-term; dark orange for long-term) macrophages respectively (c-f) Closed bars and symbols represent CO (light grey for short-term; dark grey for long-term) and AD (light red for short-term; dark red for long-term). Dots represent individual participant values. mRNA (normalized to GAPDH) expression was measured with RT-qPCR. The Friedman ANOVA was used to compare within-group differences, while the Kruskal-Wallis test (paired groups) was used to assess between-group differences (p*<0.0167 (p/n, assuming n = 3 comparison). Supplemental Fig. 2. Sex or APOEε4 genotype does not modulate APOE synthesis in short- and long-term Mo-MФs. Effect of sex on APOE secretion levels in (a) FCS and (b) AS supplemented Mo-MФs cultures from female-AD (n=10) vs. -CO (n=11) and male-AD (n=11) vs –CO (n=5). Genotype effect on APOE secretion levels in (c) FCS and (d) AS supplemented Mo-MФ cultures from APOEε4(+)-AD (n=10) vs. -CO (n=6) and APOE ε4 (-)-AD (n=11) vs –CO (n=10). (a-d) Closed bars and symbols represent CO (light grey for short-term; dark grey for long-term) and AD (light red for short-term; dark red for long-term). Dots represent individual participant values. mRNA (normalized to GAPDH) expression was measured with RT-qPCR. The group differ [file 12979_2023_376_MOESM2_ESM.pptx]
